# Outcomes of guidelines from health technology assessment organizations in community-based primary care: a systematic mixed studies review

**DOI:** 10.1017/S0266462324000370

**Published:** 2024-11-14

**Authors:** Ashkan Baradaran, Raymond Tolentino, Roland Grad, Isabelle Ganache, Geneviève Gore, Samira Abbasgholizadeh Rahimi, Pierre Pluye

**Affiliations:** 1 Department of Family Medicine, McGill University, Montréal, QC, Canada; 2 Institut national d’excellence en santé et en services sociaux (INESSS), Montréal, QC, Canada; 3 Schulich Library of Physical Sciences, Life Sciences, and Engineering, McGill University, Montréal, QC, Canada; 4 Lady Davis Institute for Medical Research, Jewish General Hospital, Montréal, QC, Canada; 5 Mila-Quebec Artificial Intelligence Institute, Montréal, QC, Canada; 6 Faculty of Dental Medicine and Oral Health Sciences, McGill University, Montréal, QC, Canada

**Keywords:** health technology assessment, community-based primary care, guidelines, health outcomes, systematic mixed studies review

## Abstract

**Background:**

Health technology assessment (HTA) organizations generate guidelines to inform healthcare practices toward improved health outcomes. This review sought to identify and classify outcomes of guidelines from HTA organizations within published research.

**Methodology:**

We performed a systematic mixed studies review of empirical studies that (a) referred to a published guideline from an HTA organization and (b) reported an outcome resulting from a guideline. We searched the published literature in English or French within seven databases. Outcome types were classified within five dimensions of an existing framework for online health information (e.g., relevance, cognitive/affective impact, and use). Subdimensions were inductively developed. A two-phase sequential data synthesis was performed. Phase 1: a hybrid deductive–inductive thematic analysis identified the types of outcomes and displayed their relationships on a concept map. Phase 2: descriptive statistics were tabulated by the type of outcome.

**Results:**

A total of 6,719 records were retrieved through searches on 6 February 2023. After screening, we included 120 observational studies (twenty-one qualitative, ninety-four quantitative, and five mixed methods). Phase 1 identified twenty-nine types of outcomes. The most frequently reported outcomes were within the organizational dimension (reported in ninety-four studies). The most common subdimensions were “Referrals” (thirty-eight occurrences), the “Quality of Prescriptions” (fifteen occurrences), and the “Quality of Diagnosis” (eight occurrences). For Phase 2, we could only generate descriptive statistics on seventeen outcomes. These were almost equally distributed among positive, neutral, and negative effects. Our results contribute to knowledge about the outcomes of HTA guidelines and options for documenting and measuring them in future evaluations.

## Introduction

### Rationale

The purpose of this review is to explore and measure the outcomes of guidelines from health technology assessment (HTA) organizations in community-based primary care (CBPHC). This is critical because HTA organizations strive to be useful and prove their usefulness. They also need to be transparent and accountable for their processes and to sustain funding. They need to justify their existence. Health technology is defined as tests, devices, medicines, vaccines, procedures, and systems developed to prevent, diagnose, and treat health conditions, promote health, provide rehabilitation, and organize healthcare delivery. HTA refers to multidisciplinary methods that use specific techniques to define the value of health technology (how technology is valuable) at different points in its life cycle (1). Furthermore, HTA aims to inform decision makers and policy makers to promote equitable and efficient health services and policies. Governments around the world are implementing and sustaining HTA organizations to improve the quality of healthcare, for example, clinical decision making, and improve health-related policies. These leading organizations conduct investigations funded by their respective governments and publish their results as HTA knowledge products, taking the form of guidelines, reports, patient education handouts, or educational videos.

In this study, we did not focus on guidelines that guide how to conduct HTA but rather on guidelines produced by organizations that conduct HTA. Clinical practice guidelines (CPGs) provide recommendations aimed at enhancing patient care, grounded in a systematic evaluation of evidence and an analysis of the potential benefits and harms associated with various treatment options (2). These guidelines do not prescribe a universal treatment strategy; instead, they assess the quality of pertinent scientific studies and evaluate the expected benefits and harms of specific interventions. Such assessments empower healthcare professionals to tailor treatment decisions to the individual preferences and needs of patients (2). The purpose of CPGs is to summarize and appraise the available evidence so that they can contribute to clinical decision making (3). If done rigorously, they can translate complex research findings for clinical practice and potentially improve the quality of care and outcomes. However, there are many challenges in the development of the CPGs, with limitations such as low-quality systematic reviews, conflict of interest, and lack of involvement of stakeholders (3). These challenges endanger the quality and trustworthiness of the CPGs (3).

In terms of context, this study mainly focused on CBPHC in Organisation for Economic Co-operation and Development (OECD) countries. CBPHC encompasses a wide range of primary prevention and primary care services, such as health promotion, disease prevention, diagnosis, treatment, and management of illness, rehabilitation, and end-of-life care (4). Health care is provided in a wide range of settings in CBPHC by nurses, social workers, pharmacists, dietitians, public health practitioners, physicians, and others (4). In some OECD countries such as Canada, the majority of health care expenditures are on family medicine and general practice, and the number of family physicians (FPs) is higher than all other specialties combined (5). Due to historical, political, and cultural factors, there is significant variation in how the OECD countries’ health systems have evolved (6). Thus, the use of HTA, health-related decision/policy-making processes, centralization, and regulations are important differences in OECD health systems. An international agreement over aspects of HTA methodologies and decision making exists; however, the use of HTA tends to reflect local circumstances, including necessities, financing and service provision arrangements, policy objectives, and the level of influence and control of decision/policy makers (6).

Worldwide, approximately 200 renowned HTA organizations play a pivotal role in their local healthcare systems. These members of the International Network of Agencies for Health Technology Assessment (INAHTA), Health Technology Assessment international (HTAi), Health Technology Assessment Network of the Americas (RedETSA), European Network for Health Technology Assessment (EUnetHTA), and HTAsiaLink are dedicated to promoting better health outcomes. Their efforts focus on preventing, diagnosing, and treating health conditions, providing rehabilitation, and organizing healthcare delivery (7). Outcomes of professional societies’ CPGs have been extensively evaluated since the emergence of the Evidence-Based Medicine movement in the 1990s. However, there are few studies on the outcomes of guidelines from HTA organizations. Such studies could focus on outcomes such as user satisfaction, information use, and impacts on health services and patient health in CBPHC. This suggests the need for a systematic review to explore and measure outcomes associated with guidelines produced by HTA organizations.

### Theoretical foundations

This study is based on two main conceptual frameworks. For the lifecycle of knowledge products, we use the knowledge-to-action model (8). The Knowledge-to-Action model is a conceptual framework that describes how to translate research findings into practice or policy and was designed to facilitate the conversion of knowledge into appropriate actions, thus improving healthcare outcomes. In order to gather all possible health outcomes and understand their relationship, we use the outcomes suggested by the Online Health Information framework (9). This framework includes both positive and negative health outcomes associated with primary care health information and explains how different factors can influence health outcomes.

### Objective and review questions

The present systematic mixed studies review aimed to explore and measure outcomes of text-based guidelines from HTA organizations relevant to CBPHC. We gathered qualitative, quantitative, and mixed methods studies to derive conclusions for health services and HTA organizations to inform decision makers and policy making.

With respect to the primary care-related population, including managers, practitioners, patients, and caregivers, our specific review questions are as follows:

- Q1 (qualitative): What are the types of outcomes of guidelines from OECD-based HTA organizations as reported in relevant qualitative, quantitative, and mixed methods studies?
- Q2 (quantitative): To what extent do such guidelines influence these outcomes?

Common outcomes that we considered in this study were user satisfaction, the use of information within administrative and clinical activities, and the improvement of health services or patient health, that is, mental, physical, and social well-being. In addition to these objectives, we analyzed literature reviews and reference textbooks to identify existing conceptual frameworks or theoretical models explaining these (and others) types of outcomes and their relationships.

## Methods

This mixed studies review was developed using the Toolkit for Mixed Studies Reviews (10) and reported following the Preferred Reporting Items for Systematic Reviews and Meta-Analyses (PRISMA) (11). The protocol is registered in PROSPERO (CRD42022297183) (12).

### Eligibility criteria

The inclusion criteria were as follows: (a) all types of empirical studies (coherent research question/objective, methods including data collection and analysis, and results) using qualitative, quantitative, and mixed methods involving, (b) HTA organizations (including members of INAHTA, HTAi, RedETSA, EUnetHTA, and HTAsiaLink) in the OECD countries, (c) which developed and used CBPHC guidelines, including CPGs, and excluding not guidelines on how to conduct HTA), and (d) leading to any type of outcome (e.g., user satisfaction, use of information, health outcomes, and outcomes affecting health or social services in primary healthcare). We did not look for specific interventions or comparisons.

The exclusion criteria were as follows: (a) irrelevant studies, that is, not in the primary healthcare context (not on family medicine, FPs, or general practitioners); (b) studies not focused on medical guidelines from HTA organizations or not focused on the use or outcomes of HTA knowledge products (e.g., studies on the development of CPGs by specialty societies); (c) studies lacking outcomes such as reported impacts, effects, benefits or harms; and (d) studies lacking empirical data, data collection, methods, or results, such as reviews, editorials, protocols, letters, position papers, and program descriptions. Reviews were marked and retrieved to identify conceptual frameworks and theoretical models.

Reasons for excluding studies are shown in Appendix 1 of the Supplementary Material. As discussed before, one of the main aims of HTA is cost-effectiveness. However, studies focusing on how guidelines reduce or increase costs were beyond our expertise and therefore excluded (13).

### Information sources

Published literature was retrieved through searches of MEDLINE, Embase, Cochrane Central Register of Controlled Trials (CENTRAL), CINAHL, and PsycINFO from their inception to 6 February 2023 (see search details in Appendix 2 of the Supplementary Material).

### Search strategy

**Figure 1.**
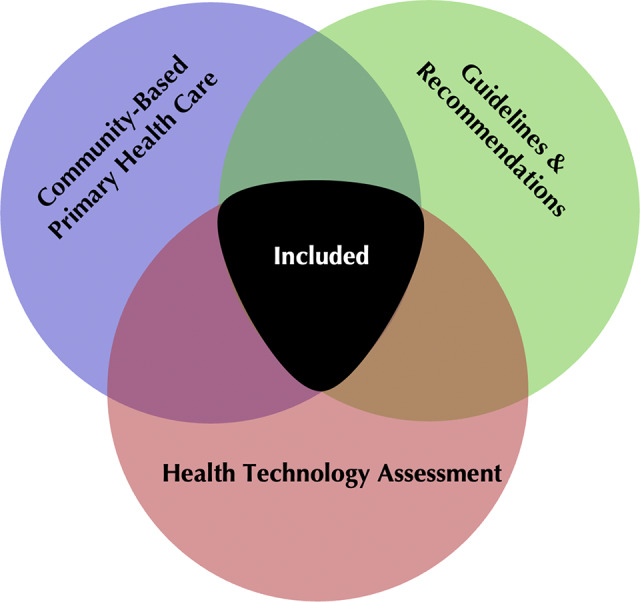
Venn diagram demonstrating the main concepts used in the search strategy. Each circle corresponds to a key concept. The common area which will be included in our review is shown in black.

A specialized librarian (G.G.) developed and executed database search strategies to gather published information on knowledge translation in HTA organizations (14). An initial set of relevant search terms was agreed upon, and additional terms were added throughout the iterative search process to ensure a comprehensive review of available literature. Search terms included primary health care, HTA, and knowledge translation (see the detailed search strategies in Appendix 2 of the Supplementary Material). Figure 1 displays the key concepts used in the search.

The search was limited to English or French language studies after 2008 up to 6 February 2023. We decided to limit our search to post-2008 due to the international agreement signed that year, which established the first comprehensive definition and methodology for HTA by international HTA agencies. Based on the three concepts shown in Figure 1, we gathered a list of 122 HTA organization names. Previous studies have used the OECD countries to analyze performance and make comparisons in health status at an international level (15;16). Accordingly, due to the breadth of the “practice guidelines as topic” heading, we narrowed it down using OECD country names (see Appendix 2 of the Supplementary Material).

Regarding grey literature, we searched Google Scholar using a simplified version of our search strategy. We decided to screen the first fifty pages of results, or until no seemingly relevant record was on a page. EndNote® 20 (17) was used as a reference manager. Citation tracking of included references was performed using Scopus.

### Selection process

Results from all databases were gathered in an EndNote library, and duplicates were removed according to a method described by Bramer et al. (18). Once documents were identified and unified, titles, and abstracts were screened by two authors (A.B. and R.T.) separately using specialized software (Rayyan). At this stage, we only excluded studies that both reviewers had excluded.

Once full texts were identified and collected, they were screened by two authors (A.B. and R.T.) separately using Rayyan, a specialized open-source software. At this stage, we only excluded studies that both reviewers had excluded. Then, selected full-text publications on which the two reviewers had disagreements were discussed in a meeting to reach an agreement for inclusion. The reviewers resolved all divergences through conversation and involved a third researcher (P.P.; third-party arbitrage) when they could not reach a consensus.

For each selection step, Cohen’s Kappa was calculated and interpreted to estimate the inter-rater agreement (19). To identify additional studies that could be considered, we performed iterative citation tracking using Scopus up to saturation (i.e., repeated until no additional relevant studies were found) (20). In the citation tracking process, we screened the references of included papers and records that cited the included studies. PRISMA flow diagram was used to illustrate the selection process.

### Data collection process

The results of all included studies were extracted in numerical, tabular, and textual formats. Two authors extracted the data separately (A.B. and R.T.). Study characteristics and outcome-related data were gathered into a predesigned Microsoft Excel spreadsheet and prepared for further analysis. Qualitative data (extracts of full texts) were collected using NVivo. We grouped the studies according to their outcomes, study design, and type of comparison.

### Data items

We used a data extraction form based on the Cochrane Effective Practice and Organisation of Care (EPOC) data collection checklist (21). Details that were extracted included the first author’s name, year of publication, data collection period, country, intervention, control, participants, setting, methods, and outcomes (such as patient outcomes, utilization, quality of care, adverse effects or harms, resource use, physician outcomes, knowledge, attitudes, performance, satisfaction). Specific details, including the study design, sample size, sampling procedures, and data collection procedures, were then collected. All available types of outcomes and related information were also extracted. The search and data extraction were not restricted to a specific type of stakeholder; however, since most of the studies we included reported outcomes related to patients and FPs, we were only able to report findings related to these two types of stakeholders.

### Critical appraisal (MMAT)

We used the Mixed Methods Appraisal Tool (MMAT) as a checklist for both appraising and describing studies included in our systematic mixed studies review. All included studies were assessed independently by two authors (A.B. and R.T.) for methodological validity using the MMAT (22). Any disagreement between the two reviewers was resolved through discussion. A third researcher (P.P.) was involved when a consensus was not reached. Finally, we calculated scores (percentages) and put them in tables along with the number of responses to each question to show whether there was heterogeneity in MMAT responses and scores among the included studies. No study was excluded based on the appraisal.

### Effect measures

Effect measures varied according to the data available, expressed as means, proportions, correlations, standardized mean differences, risk and odds ratios, incidence rates, and ratios. We did not perform a meta-analysis because we had heterogeneous outcomes for various conditions in various contexts.

### Synthesis methods

In order to discover and examine the outcomes of HTA guidelines, a two-phase sequential data synthesis was performed (23). The qualitative section of this study aimed to explore the outcomes of guidelines from HTA organizations for CBPHC. For the second phase, we provided descriptive statistics and reported knowledge gaps.

Regarding Q1 (qualitative): To the best of our knowledge, there is a lack of literature on the outcomes of HTA organizations; therefore, a hybrid thematic analysis was most appropriate to describe the outcomes and allow new insights from the literature to emerge (inductive analysis) (24). We also used the Online Health Information framework (9) to organize and recognize the main themes and dimensions and form concept maps (deductive analysis). For the first phase of the analysis, we conducted a qualitative synthesis using hybrid thematic analysis and concept mapping (identifying outcomes – themes – and their relationships to produce a map). The included full texts were reviewed by two independent reviewers. Codes were derived from the texts. Then, the reviewers compared their codes and reached an agreement on the final coding. Titles, abstracts, codes, and relevant parts of texts from the included studies, and the source (guidelines being discussed) were all gathered into memos (linked to full texts) in NVivo. After the memos were created, two reviewers (P.P. and A.B.) labeled the codes, sorted them, clarified their meaning, and created themes based on agreements reached through discussions.

Regarding Q2 (quantitative): We found two types of quantitative studies: (a) quantitative nonrandomized studies and (b) quantitative descriptive studies, comparing the outcomes pre- and postpublication, or describing the outcomes post-publication. Then, we analyzed the extracted data according to three primary care sub-populations: health care organizations, FPs, and patients.

#### Hybrid thematic analysis

A hybrid thematic analysis was the method of choice as we had pre-existing frameworks along with the data from the included studies.

As the data were gathered, read, and re-read by authors, the structure of codes was polished. Our iterative process started as the codes were created. Some codes were merged, and some were edited and changed. We used paraphrasing and summarizing in our memo files (linked with the full texts in NVivo). In the next stage, we confirmed and legitimated themes. The sub-themes were grouped based on the Online Health Information framework from Pluye et al. (9); however, subthemes related to organizational outcomes could not be grouped. Those subthemes were used to create new themes. For the first three dimensions (i.e., relevance, cognitive/affective impact, and use), we identified subthemes as facilitators and barriers related to health care providers or patients. For the fourth and fifth dimensions, that is, individual and organizational health outcomes, we grouped them based on direction (positive, negative, or neutral/other) for health care providers and patients. A negative outcome occurs when the guideline fails to achieve its specific intended change or goal. This includes situations where an objective was set, and despite the implementation of the guideline, this objective was not met. The key aspect of a negative outcome is the failure to achieve a goal, reflecting a discrepancy between the desired and actual effects of the guideline. We consider an outcome to be neutral when the implementation of a guideline does not result in any change, including, where no specific change was anticipated or targeted. It is important to distinguish this from negative outcomes by emphasizing that neutral outcomes refer to situations where the absence of change aligns with the lack of a specified goal for change, rather than being indicative of a failure to achieve an intended effect. We relied on the reviewers’ discretion in determining whether the reported changes were in line with the guidelines’ goals. Any conflict between authors (A.B. and P.P.) during the coding and creation of subthemes, dimensions, or details was resolved by a third author (R.G. or S.A.R.).

We classified the outcome as “other” when we thought that the reported outcome belonged to a specific dimension, but we could not group it under any of the existing sub-dimensions. When the qualitative outcomes were identified, we created a table with all the dimensions (themes), types of outcomes (subthemes), and the definition of each outcome. We provided clear definitions as described by the International Organization for Standardization (ISO) (25). Then, to summarize our findings, we regrouped similar and relevant subthemes in each dimension and described them together with examples. Eventually, to report the results of each dimension, we gathered the subthemes and merged them to form more generalizable statements, and we reported them along with some examples from the subthemes.

#### Concept mapping

The subthemes, themes, and dimensions were arranged into a concept map according to criteria by Novak and Gowin (26). The arrows indicate the relationships between dimensions. The position of the concepts on the map follows a hierarchy (from top to bottom), and the map shows a meaningful connection between segments of the concept hierarchy.

#### Descriptive statistics

**Figure 2.**
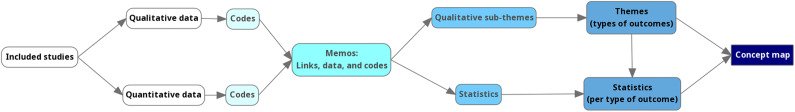
Visual display of the qualitative and quantitative analysis from included studies to the concept map.

After separating the extracted quantitative data, we included passages from the text explaining the quantitative outcomes and our interpretation of these outcomes. We grouped the outcomes based on qualitative dimensions, as shown in Appendix 3 of the Supplementary Material. We reported the results of each included study and created a table to show the direction of each effect for vote counting (Appendix 4 of the Supplementary Material). To summarize and clarify each step taken from the included studies to reach the concept map, we designed a visual display of the analysis (Figure 2).

## Results

### Study selection

**Figure 3.**
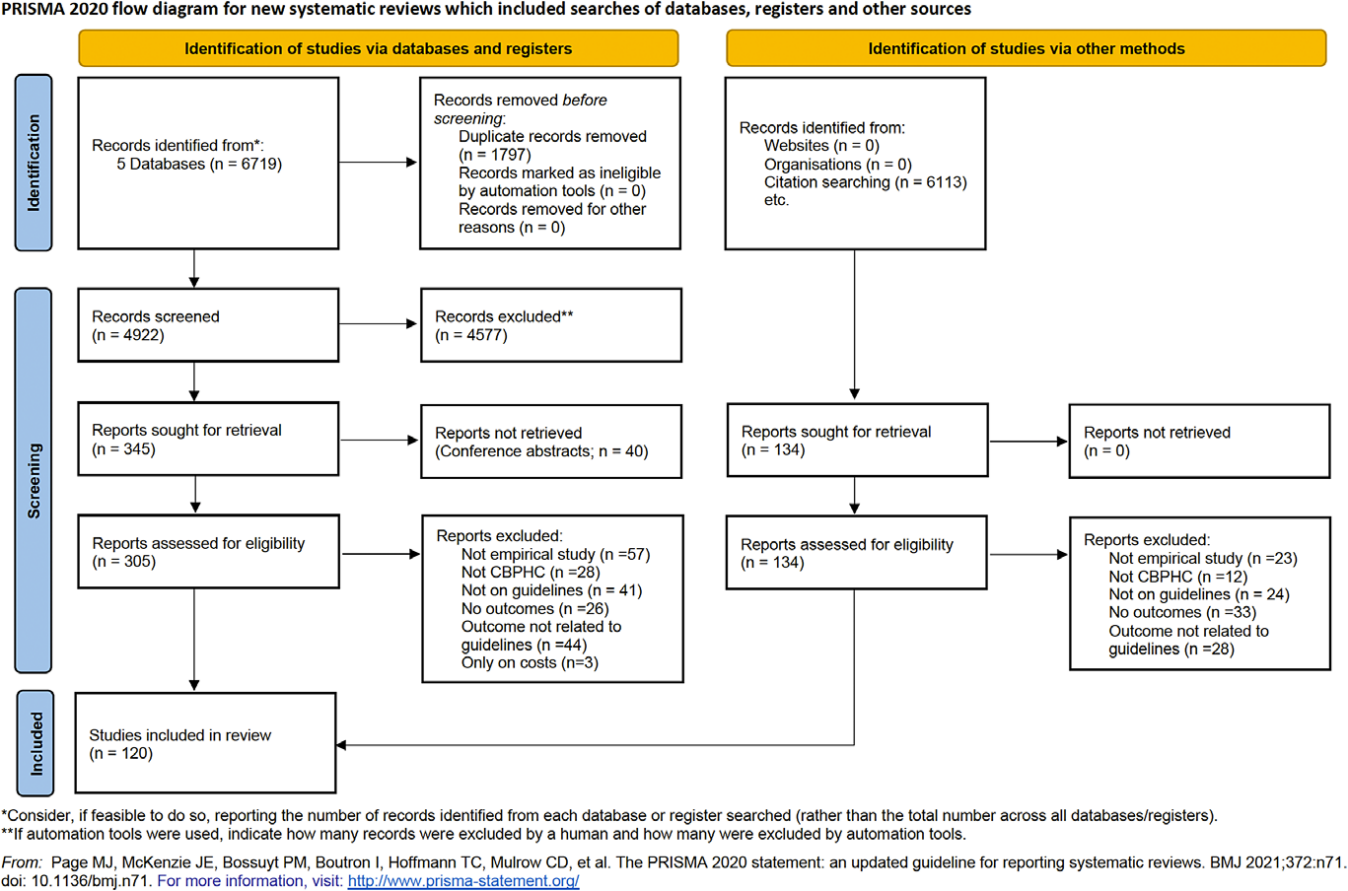
PRISMA flow diagram demonstrating the flow of the information throughout the selection process.

6719 studies were initially identified from the search on 6 February 2023 (Appendix 5 of the Supplementary Material). After deduplication, of 4,922 studies screened at the level of titles and abstracts, 305 were eligible for full-text screening. The flow of the information through different phases of the review, reasons for exclusion, and the number of studies removed with each reason are shown in Figure 3 and Appendix 1 of the Supplementary Material. Citation tracking of the included studies took eight rounds until saturation and eventually resulted in fourteen additional studies. The grey literature search did not result in any included study.

### Study characteristics

This mixed studies review included 120 studies; five mixed methods studies, twenty-one qualitative, and ninety-four quantitative studies investigating the outcomes of guidelines from HTA organizations relevant to CBPHC. The included studies are further described in Appendix 6 of the Supplementary Material. Most studies (*N* = 93, 77.5 percent) were from the United Kingdom (UK) and concerned National Institute for Health and Care Excellence (NICE) guidelines. Others were conducted in the United States (*N* = 6), Sweden (*N* = 5), Netherlands (*N* = 5), Norway (*N* = 4), France and Spain (*N* = 2 each), Finland, Germany, and Denmark (*N* = 1 each). The studies provided a diverse and comprehensive coverage of all aspects of the guidelines. Most of them (*N* = 101) had retrospective designs and compared outcomes pre- and postpublication. No study compared having a guideline with not having a guideline.

### Qualitative synthesis

#### Qualitative hybrid thematic analysis

From the 120 included studies, 312 codes were identified. Codes were gathered along with the corresponding parts of texts in 120 memos in NVivo. Based on memos, 213 subthemes were developed. Then, we created five main dimensions and assigned each subtheme to a corresponding dimension (see Appendix 4 of the Supplementary Material). The first four dimensions are derived from the outcomes of Online Health Information (9), while the last dimension is novel and derived from themes suggested by the data in an inductive manner. Nearly half of the subthemes (108/213; 51 percent) reported positive outcomes (defined as any observed or supposed improvement; e.g., when a guideline recommendation improved health status, or physicians believed a guideline was helpful). Of 213 subthemes, 73 (34 percent) subthemes were negative (defined as any observed or supposed deterioration, or in some cases, not achieving an expected outcome or change) while 32 (15 percent) were neutral (defined as no change or not categorizable as positive or negative). In the following section, we will report on the five dimensions and types of outcomes under each dimension. The five themes and twenty-nine subthemes, along with their definitions, are presented in Appendix 3 of the Supplementary Material.

##### 1. Relevance (dimension 1)

Relevance can be determined based on whether the guidelines were relevant to the study context or situation. In the following example, the terminology used in guidelines is uninformative (therefore irrelevant) for patients: *“Many also felt that non-specific Lower Back Pain was an unfamiliar term that lacked information and was thus unsatisfactory for patients.”* (p. 1844) (27). In addition, guidelines can be irrelevant when they do not consider life stages: *“The younger adults perceived older adults’ consumption of daily bottles of wine as concerning, whereas the older adults viewed younger people’s binge drinking on weekends as problematic.”* (p. e187) (28). We developed fifteen subthemes related to the relevance of guidelines. Twelve were from FPs, and three were from patients. Moreover, the following subthemes reveal four components of relevance:

*1.1. Relevance-related patients’ characteristics*: Guidelines should fit patients’ characteristics (including age and language). For example, “CPG offer overly standardised treatment and not tailored to patients’ characteristics.” (p. 349) (29).

*1.2. Relevance-related comprehensiveness*: Guidelines should offer clear recommendations for all pertinent conditions and contexts. Guidelines are not relevant when important information is missing. For example, guidelines can be perceived as irrelevant when they do not mention differential diagnoses or when they lack clear recommendations.

*1.3. Relevance-related feasibility*: Guidelines can be irrelevant when resources are scarce, and recommendations cannot be applied. *“GPs described such primary care mental health services as scarce resources with long waiting times, so they felt the need to reserve these interventions for those patients who they felt had more overt mental health symptoms, rather than a condition such as IBS…”* (p. 5) (30).

*1.4. Relevance in situations of uncertainty*: Guidelines can be considered more relevant when there is clinical uncertainty. *“Primary care practitioners in general, … were positive about guidelines and used them where there was clinical uncertainty, often in short formats…”* (p. e722) (31).

##### 2. Cognitive or affective impact (dimension 2)

When guidelines are relevant to the situation, they are more likely to be read by FPs and patients. For example, a change in guidelines can cause confusion among physicians: *“And I think because a lot of those guidelines and rules change over time, there’s just a lot of confusion. So I think it is kind of this squishy black hole to a lot of primary care doctors as far as the nitty gritty details.”* (p. 10) *(32).* And sometimes guidelines can be reassuring: “*…it’s also nice knowing that if you are at all worried they’ll definitely be seen within those 2 weeks.”* (p. 4) *(33).* We developed thirty-three subthemes related to cognitive or affective impact (twenty-five from FPs, seven from patients, and one from both). Our analysis highlights the following:

*2.1. Understanding*: Changes in guidelines can cause confusion among FPs, and literacy can affect patients’ understanding of the guidelines. *“They criticised difficult terms such as “chronic” and “pneumonia”, which sound foreign in the Finnish language.”* (p. 216) *(34).* However, guidelines can help patients understand their disease.

*2.2. Learning something new*: Guidelines can improve FP knowledge and change their perception. “*The guidelines clearly reflect the practitioner’s perception of the clinical value of a throat swab…”* (p. 7) *(35).* However, they sometimes fail to improve FP knowledge; for example, regarding diagnostic tests: “*The GPs expressed a belief that the clinical picture was sufficient for diagnosis in typical cases.”* (p. 1) *(36).* And regarding contraindications: “*Among 2009 Home Blood Pressure Monitoring users, only 44 percent declared knowing of its contraindications, but in actual fact had very little knowledge of them.”* (p. 2109) *(37).* Guidelines fail to improve knowledge when FPs have too many guidelines to read. “*Most GPs described how they received a large number of guidelines each week, limiting the time available to read all in detail and, as a result, did not feel that they had full knowledge of guidelines for perinatal depression”* (p. 7) (38). FPs’ knowledge can depend on their patients’ conditions. *“GPs with more frequent involvement in solid tumor follow-up had higher LE (late effects) awareness scores….”* (p. 364) (39). Guidelines can help patients learn about their condition by improving engagement. “*Participants already engaged in some form of self-management, either self-learned from experience or disseminated via social networks.”* (p. 6) (40)

*2.3. Validation*: Guidelines can enhance awareness of treatment effectiveness and validate current practice. *“… NICE guidelines might enhance awareness of effective and ineffective treatments, validating existing practices.”* (p. 1846) (27)

*2.4. Reassurance (trust)*: Some guidelines are reassuring for FPs. “*… I think it’s also reassuring to know that treatment will be instigated within a certain period of time.”* (p. 4) (33). However, they have doubts about some recommendations. “*… some GPs expressed doubt about the evidence-base for these interventions…”* (p. 5) (30). Patients are more likely to trust guidelines from credible sources (34).

*2.5. Remembering*: According to the Online Health Information framework (9), in some instances, the guideline could help stakeholders recall the health information they already knew. None of the included studies reported outcomes related to memory.

*2.6. Motivation*: Too many guidelines can reduce FP motivation. *“… there’s millions of them and it’s absolutely impossible in normal general day to day practice to be au fait with them all.”* (p. 4) (30)

*2.7. Satisfaction or dissatisfaction*: Some FPs are satisfied with guidelines “*expressed their appreciation of the guidelines and claimed that they followed them…”* (p. 195) (41). Moreover, some patients believe that guidelines ensure the quality of care. On the other hand, some FPs believe that guidelines can be a burden and a threat to their autonomy (29), and some patients are not satisfied with the content of guidelines (28).

*2.8. Mispresentation*: None of the included studies reported outcomes related to mispresentation (misinformation or mispresentation of information).

*2.9. Disagreement*: Disagreement with one recommendation can result in some FPs not using a guideline (42).

*2.10. Guideline potentially harmful*: No study reported outcomes related to guidelines being potentially harmful.

*2.11. Willingness to discuss sensitive information*: Some FPs might avoid discussing and implementing deprescribing guidelines (43).

##### 3. Use for patients or practice (dimension 3)

Information use was an important outcome of HTA guidelines. An essential type of use was instrumental use, which means that the guideline was used to do something differently (a change in practice). For example, physicians can extend their practices using the guidelines: *“However, some practitioners thought that the NICE guidelines might enhance awareness of effective and ineffective treatments, validating and/or slightly extending existing practices.”* (p. 1846) (27). We developed forty-two subthemes related to use (thirty-three from FPs, seven from patients, and two from both). We identified the following types of information used in association with guidelines:

*3.1. Conceptual*: Guidelines are useful in times of uncertainty, and they can increase FP confidence in tests and treatments.

*3.2. Legitimating*: FPs can use guidelines to support and justify their clinical decision making. *“Also used as safeguard to avoid patient complaints and litigation.”* (p. 13) (29). Therefore, guidelines can be protective in that matter. Patients can use guidelines to communicate with their physicians and justify their problems.

*3.3. Instrumental*: Guidelines can affect FP practice by changing protocols, changing standards of treatment, “… *slightly extending existing practices.”* (p. 1846) (27); but sometimes guidelines are not associated with a change in practice. *“GPs in all categories made similar decisions for each case-vignette, no matter which guideline was applied (or no guideline applied).”* (p. 6) (44) and *“I’ve read all that but it’s still very hard to go away from something that was drummed in.”* (p. 4) (30). Guidelines can also affect patients care seeking behavior (such as consultation habits).

*3.4. Symbolic*: From the FP perspective, guidelines are considered acceptable rules and references that support their decision making, for example, some FPs mentioned *“… citing national recommendations, or safety research, as a form of back-up during conversations…”* (p. 3019) (43). They also discuss new guidelines with their peers in meetings, for example, some FPs *“… discussed the clinical guidelines in formalized meetings and made informal oral agreements to make a change based on guideline recommendations.”* (p. 683) (45). Guidelines can also inform patients in clinical encounters; for example, some FPs mentioned that guidelines “*… helped them to back up the key messages they delivered regarding the treatment and management of Osteoarthritis…”* (p. 5) (40). Furthermore, *“patients consider guidelines to include instructions or standards for professionals, information given by health professionals to patients, and material to protect and promote the interests of patients.”* (p. 213) (34).

##### 4. Individual patient health outcomes (dimension 4)

Individual patient health outcomes are directly related to patient health and not the health care system. For example, anticoagulation guidelines are associated with a decrease in hospitalizations for thromboembolic conditions: *“Increased Direct oral anticoagulant prescribing was associated with a slight decline in admission for thromboembolic conditions.”* (p. 1) (46).

We developed twenty-four subthemes related to individual patient health outcomes (sixteen from patients, seven from FPs, and one from both). Moreover, the subthemes revealed the following outcomes:

*4.1. Health improvement or harm*: Guidelines are associated with improved health outcomes such as decreased admissions or risk of complications, improved control of conditions, detection rates, and prescriptions. For example, anticoagulation guidelines are associated with *“… emergency admissions for bleeding complications.”* (p. 4) *(46)*; and after the implementation of malnutrition guidelines, *“the proportion of individuals at risk of malnutrition reduced over time…”* (p. 1) (47). However, the outcomes can vary depending on the population, and guidelines may or may not be associated with a change in health outcomes; for example, *“Thirty-five percent of patients achieved a target HbA1c of <6.5 percent compared to 25 percent in England. Applying the NICE target for blood pressure (≤140/80 mmHg), 54 percent of patients reached this target comparable to 60 percent in England. Slightly less patients were categorised as obese (>30 kg/m^2^) in Ireland (50 percent, n = 1,060) compared to Scotland (54 percent).”* (p. 1) (48)*;* and for colorectal cancer patients, *“the 5-year survival rates in the pre-2WW and post-2WW groups did not differ significantly …”* (p. 1) (49). Guidelines can sometimes cause harm; and regarding guidelines recommending an increase in physical activity for patients with depression, some FPs said that *“… there may be some people for whom there may be a negative impact.”* (p. 16) (50)

*4.2. Increase or decrease worries*: Informing patients based on guidelines (about their risk of disease) and adhering to the recommendations (such as deprescribing medications) can cause worries for the patients. On the other hand, some patients might feel reassured knowing that their FP is adhering to the guidelines.

*4.3. Preventive care*: Meeting the targets set by the guidelines can reduce the risk of future events, such as cardiovascular disease.

*4.4. Management of a problem*: No included study reported outcomes related to the management of health problems.

##### 5. Organizational health outcomes (dimension 5)

This dimension is novel and has been added to the Online Health Information framework. One of the most studied themes in this dimension was “referrals”. For example, guidelines can improve referrals and prescriptions: “*There was an increase in specialist referrals from 24 percent to 28 percent. Median time to referral was 1.5 days. Prescribed compression hosiery declined from 20 percent before the new guidelines to 18 percent after the new guidelines.”* (p. 1) (51)

We developed ninety-four subthemes related to organizational health outcomes (eighty-eight from FPs, three from patients, and three from both). We have identified the following types of organizational health outcomes:

*5.1. Clinician–patient relationship*: Guidelines can facilitate the clinician ***–*** patient relationship and increase the quality of consultations. However, guidelines can affect FP credibility and might increase doctor-shopping by patients. For example, one FP said: “*It was a challenge, but I’d say 50 percent listened to me…and 50 percent were like, ‘I’m just going to go see somebody else.”* (p. 3019) (43).

*5.2. Referrals*: Guidelines can improve referrals by reducing delays, for example, “*This change in the referral pattern was reflected in the overall interval from referral to treatment, which decreased significantly …”* (p. e178) (49), improve referral letters (e.g., “*Cognitive screening instrument use referred to in referral letters from primary care was increased …”* (p. 274) (52)), and simplify referral (e.g., “*They included organ-related signs and symptoms with a threshold positive predictive value of ≥3 percent in order to diagnose cancer at earlier stages and to simplify referral for primary care practitioners.”* (p. 408) (53), and “*Changes in justification and quality of referrals”* (p. 6) (54)). Guidelines are associated with changes in referral patterns and diagnosis rates (e.g., for head and neck cancer “*… there was an 84 percent increase in 2ww referrals from 2009 to 2013.”* (p. 416) (55)); and for dementia guidelines, “*There was a small decrease in the overall percentage of patients receiving a diagnosis of dementia.”* (p. 275) (52); and varicose vein guidelines were “… *associated with a 112 percent increase in the number of people referred with LU (leg ulcer).”* (p. 549) (56). Referral guidelines might cause delays (e.g., the 2WW pathway increased the number of referrals and *“The extra demand has created a change in clinic availability and delays other patients from seeing a specialist via the normal avenue of referrals.”* (p. 310) (57)). Some referral guidelines are not associated with an improvement in referrals in terms of detection rates (e.g., *“The detection rate for malignancy from 2WR referrals in the present study falls well below that of previous studies in the UK”* (p. 408) (53)). FPs who thought the patient had cancer would ignore cancer referral guidelines, even when the symptoms did not match the criteria described in the guideline. Referral guidelines might or might not improve prognosis (e.g., “*There were no statistical differences in clinical staging and overall survival between 2ww and non-2ww patients.”* (p. 1) (55); *“The mortality rate was 14 percent vs 6 percent (p < 0.001) and hospitalisation rate 38 percent vs 27 percent (p < 0.001) for the 2WP vs 6WP patients, respectively.”* (p. 1582) (58)).

*5.3. Integrated care*: Guidelines might ensure uniformity of care. Referral guidelines might or might not be helpful in communications: “*Changes in communication between primary and secondary care”* (p. 6) (54), and some FPs state that referral guidelines are not useful in areas where they do not know the consultant physicians.

*5.4. Standardized care*: Guidelines can contribute to standardized and improved clinical practice.

*5.5. Quality of investigations:* Guidelines can increase appropriate investigations requested by FPs. Guidelines are associated with improvements in screening and monitoring investigations (e.g., “*…the percentage of patients who had their HbA1c level assessed increased…”* (p. 1) *(59)*). Guidelines can change investigation patterns (e.g*., “… there is likely to be a significant increase in the requirements for coronary angiography, functional imaging and CT calcium scoring.”* (p. 187) (60)). Lower investigation rates recommended by some guidelines can cause higher missed diagnosis (e.g., “*Overall, it would have only detected one quarter of the abnormal cases (8 vs 32) and would have missed five of nine children with scarring…”* (p. 1) (61)).

*5.6. Quality of diagnosis:* In terms of diagnosis, guidelines might save time, for example, *“after guidance revision, New-NICE diagnostic intervals became shorter than Old-NICE values for colorectal cancer.”* (p. 1) (62). Guidelines have an impact on FPs and can change diagnosis patterns, for example, “*Sustained reductions were found in the proportion of first-ever depression episodes treated within 12 months…”* (p. 1) (63); and when FPs where asked, “*over half felt that both NICE and QOF had made little or no impact upon their detection and clinical management.”* (p. 127) (64).

*5.7. Quality of prescriptions**:*** Guidelines can improve prescribing, for example, FPs “…*stated use of rapid antigen detection test when at least 2 Centor criteria were present and prescribed antibiotics only when rapid antigen detection test was positive.”* (p. 3) (65). Guidelines can also improve patient compliance with prescriptions.

*5.8. Quality of management of chronic disease:* Guidelines can sometimes improve the quality of management of conditions, for instance, *“Encouragingly, following publication of NICE CG 168, there has been a statistically significant improvement in the management of VV (varicose veins) in primary care…”* (p. 882) (51).

*5.9. Healthcare use (efficiency):* Guidelines can save FP time (e.g., “*A majority of GPs perceived the local guidelines as time saving.”* (p. 4) (66)). However, some guidelines can be time-consuming (e.g., regarding safety-netting guidelines a FP said that *“… it would be too time consuming to type it out every time.”* (p. e821) (67); and some FPs report that long guidelines might waste their time). Guidelines can improve efficiency by reducing the need for care (e.g*., “The proportion of individuals at risk of malnutrition reduced over time…”* (p. 1) (47) which in turn has a positive impact on healthcare use; and *“The rate of consultation subsequently decreased…”* (p. e296) (68) after the introduction of hypertension guidelines).

*5.10. Psychosocial effects:* Guidelines can have different impacts on FPs based on psychosocial factors, for example, “*the willingness to take responsibility for Hodgkin’s lymphoma survivors earlier was also associated with familiarity with guidelines on LEs (late effects) after radiotherapy”* (p. 365) (39), and “*the degree of impact of NICE on management was greater for younger GPs … and doctors in larger practices …”* (p. 127) (64).

### Concept mapping

**Figure 4.**
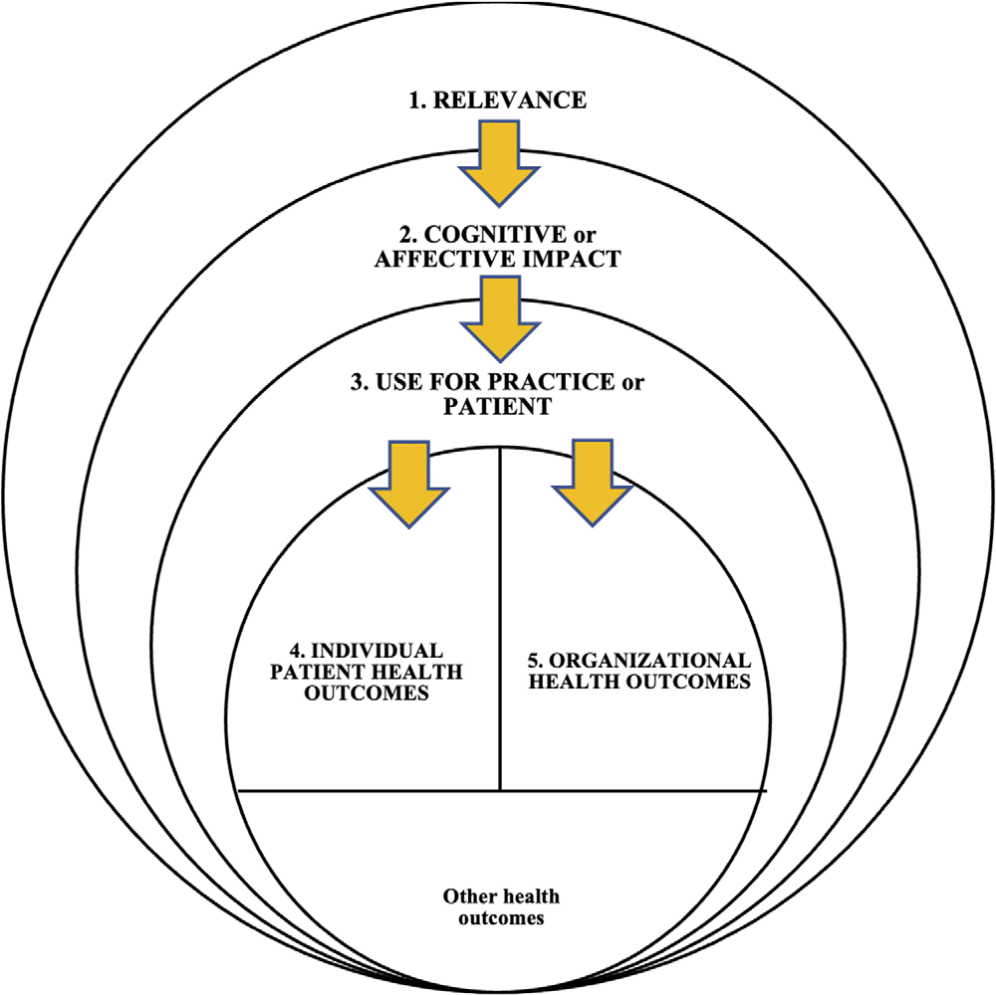
Conceptual framework representing the five dimensions of outcomes of primary care guidelines from HTA organizations.

According to the five dimensions (themes), we developed a concept map demonstrating the relationship between themes (Figure 4). This figure represents these dimensions, from relevance to health outcomes, meaning that if the results of an included study reported an outcome, the HTA product was relevant to the situation, had a positive cognitive/affective impact, was used, and had an outcome. In other words, when information derived from the HTA product:

- Is not relevant, it does not have a cognitive/affective impact, it is not used, and therefore it has no outcome;
- Has no cognitive/affective impact (e.g., learning), is not used, and has no outcome;
- Is not used, therefore it has no health-related outcome for the individual or the organization.

We also acknowledge that other types of health outcomes were not reported in our included studies. For example, population health outcomes (morbidity and mortality).

### Quantitative synthesis

As heterogeneous outcomes were reported in the qualitative phase, meta-analysis was not feasible. Thus, we report descriptive statistics for each derived dimension from the qualitative phase (Appendix 4 of the Supplementary Material).

According to our findings:

1. There are little to no (*n* = 0–1) quantitative findings for seventeen outcomes.
2. There are few (*n* = 2–4) quantitative findings for nine outcomes.
3. There are five or more quantitative findings for five outcomes.

**Figure 5.**
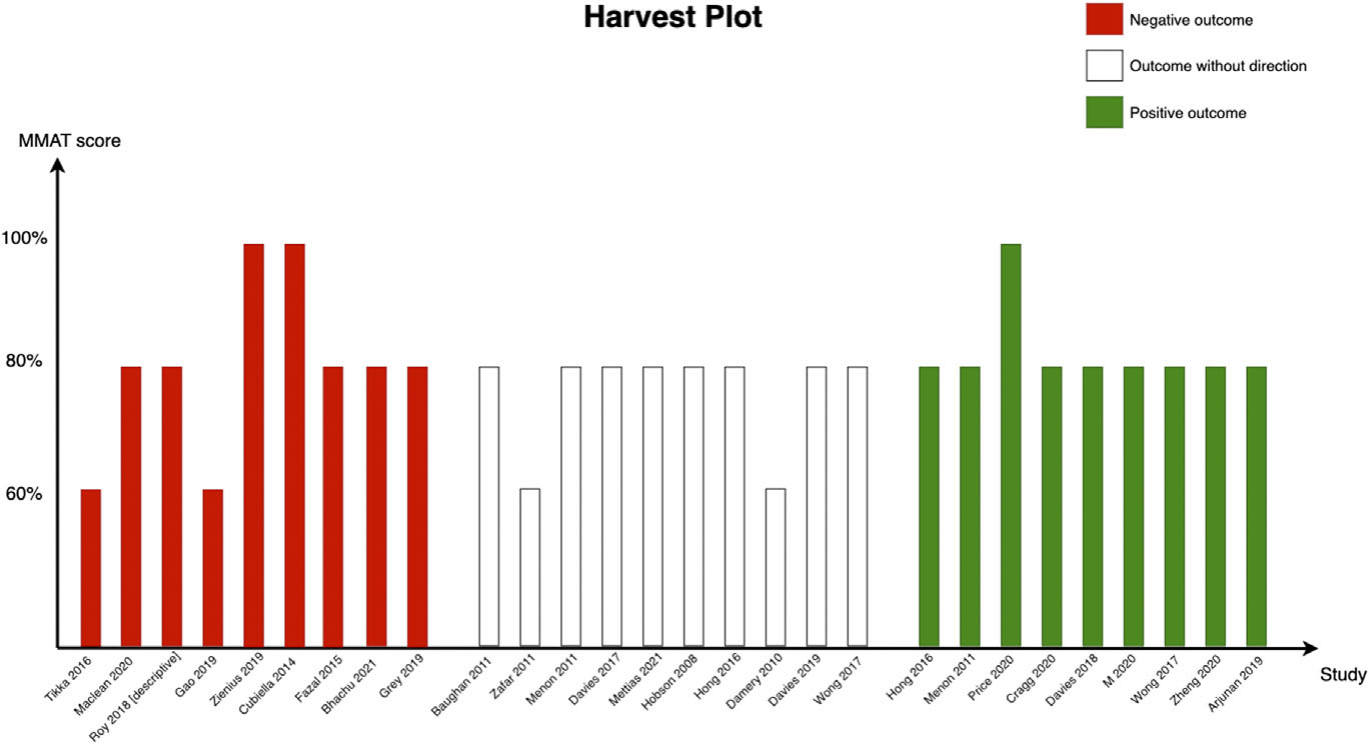
Harvest plot for referrals. Each colored bar shows one study, and the *Y*-axis shows the MMAT score of each study. As can be seen, there is no noticeable variation in the quality of studies in the positive, neutral, or negative groups.

The most frequently reported outcome was referrals (*n* = 29). With nine positive, nine negative, and eleven neutral findings, this outcome was also well-balanced. Therefore, we decided to examine the variability of the MMAT score for this outcome (Figure 5). We observed no important difference in the MMAT scores of studies in each of these three directions (comparing positive, neutral, and negative).

### Heterogeneity

We found the included studies to be clinically and methodologically heterogeneous. Regarding clinical heterogeneity, participants’ characteristics varied between studies. Data sources were also diverse; some studies used health records and databases as a source of information, and others used health questionnaires. Additionally, the included studies had different study designs. We attempted to stratify studies based on their design, participants, and outcomes to address heterogeneity. Eventually, the heterogeneity was considered to have no effect on the resulted dimensions and concept map.

### Critical appraisal

The included studies comprised qualitative, quantitative, and mixed methods studies. The results are displayed in Appendix 7 of the Supplementary Material (summary) and Appendix 6 of the Supplementary Material (complete). The majority (82.5 percent) of included studies had an MMAT score of 80 percent or higher. There was low variability in MMAT scores among the outcomes.

## Discussion

In this systematic mixed studies review, we aimed to describe and measure the outcomes of guidelines produced by HTA organizations for CBPHC, particularly family medicine/general practice. As a result of this study, we generated a list of twenty-nine types of outcomes of guidelines from HTA organizations (Appendix 4 of the Supplementary Material). We grouped outcomes into five dimensions and mapped the relationships between these dimensions (Figure 4). We developed a conceptual framework that could help us understand how HTA guidelines lead to health outcomes. Of the twenty-nine types of outcomes, only seventeen(58.6 percent) were represented in the quantitative phase. Most of the quantitative results were on organizational health outcomes (specifically specialist referral from primary care), and most of the quantitative outcomes in that dimension (organizational health outcomes) had a clear direction, whereas most of the quantitative outcomes in the first three dimensions (relevance, cognitive/affective impact, and use) did not have a clear direction (neutral).

Furthermore, as we assessed the literature on this topic, we identified gaps in knowledge. This has implications for future research. Worldwide, as there are more publications about NICE guidelines compared with other HTA organizations, most of the included studies were from the UK and about NICE guidelines. According to the previous studies and our bibliometric study done prior to writing the protocol (Appendix 8 of the Supplementary Material), most authors on this topic were U.K.-based (69). The findings of this study will provide insight into evaluation projects, particularly the evaluation of HTA guidelines. These findings are mainly focused on the outcomes documented after guidelines are published, and do not cover the production and dissemination of guidelines, nor particular suggestions stakeholders might have regarding the guidelines. By providing a detailed analysis of the outcomes of HTA guidelines, our study contributes to knowledge and opens new avenues for future research. It emphasizes the need for a more diverse geographical representation, a balanced assessment of various outcome dimensions, and an exploration of the complete lifecycle of guidelines for studying HTA guidelines. By taking this approach, we can hopefully develop a more effective set of HTA guidelines for improving the quality and effectiveness of health care.

While clinical guidelines aim to enhance patient outcomes and guide practices, their application and effectiveness might be influenced by the healthcare context in which they are implemented. In low- and middle-income countries, where resources are limited, innovative approaches such as mHealth and education via digital platforms offer promising avenues for guideline implementation (70;71). In contrast, high-income countries’ well-established healthcare systems allow clinical guidelines to be applied more widely and consistently (72). In order to bridge these disparities, we must understand the unique challenges and opportunities of each context (73). Flexibility and adaptability are key to creating guidelines that cater to diverse healthcare environments.

### The developed framework

In addition, our results helped to revise the initial conceptual framework (9) to provide a better explanation of how – in our case – guidelines can be associated with improved health outcomes (see Figure 4). The revised framework is comprised of the following aspects:

- Context: Our framework is about how guidelines can affect health outcomes in CBPHC. The framework starts with FPs and patients receiving or viewing the guidelines based on their needs. Information requirements in CBPHC diverge significantly from those in specialty practice, necessitating a distinct framework for our approach. Notably, referrals play a crucial role in the functioning of CBPHC practices.
- Use: Based on the FP and patient context, if the guideline is relevant and has a cognitive or affective impact, then it might be used.
- Information outcomes: When pertinent, a guideline may lead to improved physician or patient knowledge. Improved knowledge might change physician practice leading to improved health outcomes for patients. Moreover, guidelines do not always cause a change in practice; for instance, they can be used to reassure patients of the quality of care that they are receiving. In our framework, clinicians are considered as part of the health care system. For example, when prescriptions or referral patterns change, the whole health care system will be affected, but this is mainly through the actions of the clinician.

### Quantitative analysis

In the study’s second phase, the quantitative analysis identified thirty-six positive, twenty-seven neutral, and eighteen negative outcomes from healthcare guidelines, supporting the initiative to maximize benefits over harms (74). Positive outcomes were characterized by improved referral and diagnosis times, whereas neutral outcomes showed no significant change (e.g., in cancer detection rates), and a negative outcome was increased bleeding risks following guideline-adjusted anticoagulant prescriptions. This outcome variability suggests influences of both random variation and specific outcome characteristics. The study’s novelty lies in its comprehensive analysis of guideline outcomes, previously unexplored in the literature, employing MMAT scores and a Harvest plot to visually represent referral outcomes (see Figure 5). Despite the intent to conduct a meta-analysis on referral outcomes, heterogeneity precluded this approach. We found a few negative health outcomes related to the guidelines. Other studies have also reported potential harms associated with guidelines. For instance, guidelines can be wrong (due to problems in development) and cause harm (when implemented). Although it may seem counterintuitive, it has been mentioned in commentaries and correspondence (75;76). Studies that the guidelines are based on can be flawed (for instance, in terms of unplausible results and lack of reproducibility). Furthermore, the evidence (e.g., observation studies and low-quality evidence) used in the guideline development and the review methodology might be flawed (e.g., building upon mistakes from previous reviews). To have better oversight of guideline development, it is recommended that guideline panels include both content experts and patients (as advisors) and they all should avoid conflicts of interest (77). Furthermore, commercial industries can cause doubt and manipulate science in many ways to increase their profits (78). Therefore, agencies and organizations that produce guidelines should be accountable for their work.

## Strength and Limitations

There were several strengths to this systematic review. Firstly, the diversity of methodologies employed in this review, including qualitative, quantitative, and mixed methods studies, allowed for a more complete understanding of the impact of guidelines. This mixed-methods approach provided a holistic view, capturing measurable outcomes and experiential insights from stakeholders. Secondly, our extensive literature search, which spanned five databases and included grey literature, ensured thorough coverage of available studies, minimizing the likelihood of missing publications. Furthermore, the inclusion of studies on various guidelines from HTA organizations globally added a valuable comparative dimension, enabling the assessment of guideline effectiveness across different healthcare systems and cultural contexts. Lastly, the rigorous methodological framework used for data synthesis adhered to established standards, ensuring the credibility of our findings.

This systematic review faced several limitations. Primarily, the limited methodologies used in the included studies. We did not limit our search strategy; however, all included quantitative studies were observational or nonrandomized (pre- and postpublication comparisons), and we found no randomized controlled trials comparing any HTA guideline versus a control or comparison group involving a non-HTA guideline. Secondly, regarding the nature of the reported health outcomes in this review (individual and organizational), they did not include morbidity and mortality. Health outcomes we found can be considered as surrogates to morbidity and mortality which is why we dedicated part of the inner circle of the conceptual framework to “other health outcomes” (see Figure 4). We only included articles in English and French because the authors were proficient in these two languages. Also, we avoided searching for self-assessments published by agencies because we were concerned about the potential bias. Our objective was to maintain the highest possible standard of evidence by prioritizing peer-reviewed studies and reports, which are subject to rigorous academic scrutiny. Lastly, we excluded guidelines involving cost analysis as this was beyond the scope of the best of our knowledge.

## Conclusion

This systematic review provided a framework that explains the outcomes of guidelines from HTA organizations and their relationships. In addition, the qualitative analysis yielded a list of outcomes with examples derived from the empirical studies. Our quantitative analysis illustrated the importance of these outcomes. This review contributes to knowledge about knowledge products from HTA organizations. We uncovered extensive knowledge gaps that could be addressed in future research on the outcomes of guidelines.

## Supporting information

Baradaran et al. supplementary material Baradaran et al. supplementary material

## Data Availability

Requests for data sharing should be sent to the corresponding author: A.B. (ashkan.baradaran@mcgill.ca). The complete report of this study (with sub-themes and passages from text) will be made available upon request.

## Supplementary material

The supplementary material for this article can be found at http://doi.org/10.1017/S0266462324000370.

## Provenance statement

Not commissioned; externally peer-reviewed.
