# Supplementary material for: Outcomes of guidelines from health technology assessment organizations in community-based primary care: a systematic mixed studies review
Source: Int J Technol Assess Health Care. 2024 Nov 14;40(1):e56. doi: 10.1017/S0266462324000370 (PMC11579698; doi:10.1017/S0266462324000370)
Supplement: Baradaran et al. supplementary material [file S0266462324000370sup001.zip › Appendix 1.docx]

| **Appendix 1. Reasons to exclude studies in first and second level screening.** | |
| --- | --- |
| Title and abstract screening | Full-text screening |
| 1. The record is not a COMPLETED empirical study (has not reported methods and results). For example, exclude a review, an editorial, a protocol, a letter, a position paper, a program description. | 1. The manuscript does not report a COMPLETED empirical study (has not reported methods and results). For example, exclude a review, an editorial, a protocol, a letter, a position paper, a program description or conference abstract. |
| 2. The title or abstract suggests that the study is not in the primary healthcare context (not on family medicine, family physicians, or general practitioners). | 2. The manuscript shows that the study is not in the primary healthcare context (not on family medicine, family physicians, or general practitioners). |
| 3. The title or abstract suggests that the study is NOT focused on a guidance, a guideline, or a recommendation (from a Health Technology Assessment organisation) or is not focused on the use or outcomes of knowledge products (for example, a study on the development of a clinical practice guideline by a medical association of specialists). | 3. The manuscript shows that the study is NOT focused on a guidance, a guideline, or a recommendation from a Health Technology Assessment organisation. |
| 4. The title or abstract suggests that the study does not report outcomes (impacts, effects, benefits, pitfalls, etc.) of an intervention comprised of a guidance, a guideline, or a recommendation (knowledge product from a Health Technology Assessment organisation), or report only costs. | 4. The manuscript is not focused on the use or outcomes of knowledge products (for example, it reports the development of a guideline). |
|  | 5. The manuscript shows that the study does not report outcomes (impacts, effects, benefits, pitfalls, etc.) of an intervention comprised of a guidance, a guideline, or a recommendation (knowledge product from a Health Technology Assessment organisation). |
|  | 6. The manuscript reports only costs. |
