# Supplementary material for: Outcomes of guidelines from health technology assessment organizations in community-based primary care: a systematic mixed studies review
Source: Int J Technol Assess Health Care. 2024 Nov 14;40(1):e56. doi: 10.1017/S0266462324000370 (PMC11579698; doi:10.1017/S0266462324000370)
Supplement: Baradaran et al. supplementary material [file S0266462324000370sup001.zip › Appendix 2.docx]

**Appendix 2. Search strategies. Study design search component adapted from El Sherif et al. (1)**

**MEDLINE ALL (Ovid), 1946-**

1 Technology Assessment, Biomedical/ 10939

2 (health technolog* or hta or technology assessment*).af. 64388

3 (AHTA or "ASERNIP S" or "Australian Safety and Efficacy Register of New Interventional Procedures Surgical" or (PBAC and MSAC) or ("Australia Government" and "Department of Health and Ageing") or ("Menzies School of Public" and "University of Sydney") or "The George Institute for Global Health" or AIHTA or GOeG or "Gesunheit Osterreich GmbH" or "Hauptverband der Osterreichischen Sozialversicherungstrager" or "Association of Austrian Social Insurance Institutions" or ("University for Health Sciences" and "Medical Informatics and Technology") or "Belgian Health Care Knowledge Centre" or ("Scientific Institute of Public Health" and "Belgium") or ("Rijksinstituut voor Ziekte" and "en Invaliditeitsverzekering") or CADTH or "Canadian Agency for Drugs and Technologies in Health" or (IHE and CANADA) or ("Institute of Health Economics" and CANADA) or "Institut national d’excellence en sante et en services sociaux" or "National Institute for Excellence in Health and Social Services" or INESSS or ("Ministry of Health" and "Chile") or (("Institute of Public Health" or "ISP") and Chile) or ("Center for Drug Evaluation" and "Taiwan") or "China National Health Development Research Center" or CNHDRC or (IETS and Colombia) or "Instituto de Evaluacion Tecnologica en Salud" or ("Ministry of Health and Social Protection" and Colombia) or IETS or "Costa Rican Social Security Fund" or (CCSS and "Costa Rica") or ("Directorate of Scientific and Technological Development in Health" and "Ministry of Health" and "Costa Rica") or "Ministry of Health of the Czech Republic" or ("State Institute for Drug Control" and "Czech Republic") or DEFACTUM or ("Social & Health Services and Labour Market" and "DENMARK") or ("Institute of Family Medicine and Public Health" and "Estonia") or FinCCHTA or "Finnish Medicines Agency" or ("National Institute for Health and Welfare" and Finland) or "Assistance publique Hopitaux de Paris" or "French National Authority for Health" or "Haute Autorite de Sante" or "G BA" or (("The Federal Joint Committee" or "Gemeinsamer Bundesausschuss") and GERMANY) or IQWiG or (("Institut für Qualitat und Wirtschaftlichkeit im Gesundheitswesen" or "Institute for Quality and Efficiency in Health Care") and Germany) or "German Institute for Medical Documentation and Information" or "National and Kapodistrian University of Athens" or "National Evalution Center of Quality and Technology in S.A." or EKAPTY or ("National Organization for Medicines" and Greece) or ("National Organisation for Healthcare Provision" and Greece) or ("Institute of Pharmaceutical Research and Technology" and Greece) or "Onassis Cardiac Surgery Centre" or ("National Institute of Pharmacy and Nutrition" and Hungary) or ("Health Services Management Training Center" and Hungary) or HIQA or ("Health Information and Quality Authority" and IRELAND) or ("National Centre for Pharmacoeconomics" and "St. James Hospital") or Agenas or ("The Agency for Regional Healthcare" and ITALY) or (ASSR and ITALY) or (("Agenzia Sanitaria e Sociale Regionale" or "Regional Agency for Health and Social Care") and ITALY) or "HTA Unit in A. Gemelli Teaching Hospital" or "University Hospital A. Gemelli" or ("Agenzia Sanitaria e Sociale Regionale" and "Regione Emilia Romagna") or ("National Agency for Regional Health Services" and Italy) or "Italian Medicines Agency" or "Centro Regionale Unico sul Farmaca del Veneta" or ("Sede del Ministro" and "Ministero della salute") or "Regione Emilia Romagna" or ("Regione Del Veneto" and "Area Sanita E Sociale")).af. 70609

4 ((("Center for Outcomes Research and Economic Evaluation for Health" or "C2H") and "National Institute of Public Health") or ("Research Center for Health Policy and Economics" and ("Hitotsubashi Institute for Advanced Study" or HIAS)) or ("National Health Service" and Latvia) or ("The Institute of Hygiene" and Lithuania) or ("State Health Care Accreditation Agency" and Lithuania) or "State Medicines Control Agency of Lithuania" or (("National Center for Technological Excellence in Health" or "CENETEC") and "PAHO WHO Collaborating Center") or "Zorginstituut Nederland" or ("ZonMw" and Netherlands) or "The Netherlands Organisation for Health Research and Development" or ZINL or ("National Health Care Institute" and Netherlands) or "Erasmus Universiteit Rotterdam" or "Utrecht University" or "Norwegian Institute of Public Health" or NIPHNO or "Norwegian Institute of Public Health Norway" or "Norwegian Centre for E health Research" or "Norwegian Directorate of Health" or "Norwegian Medicines Agency" or AOTMiT or ("Administracao Central do Sistema de Saude" and "I.P.") or ("National Authority of Medicines and Health Products" and Portugal) or "Ministry of Health of the Slovak Republic" or "Comenius University in Bratislava" or "Public Agency of the Republic of Slovenia for Medicinal Products and Medical Devices" or "Ministry of Health of the Republic of Slovenia" or ("National instute of Public Health" and Slovenia) or NIJZ or ("NECA" and "South Korea") or ("National Evidence based Healthcare Collaborating Agency" and "South Korea") or ("Department of Health Convergence" and "Ewha Womans University") or AETS or "Agencia de Evaluacion de Tecnologias Sanitarias" or AETSA or "Fundacion Publica Andaluza Progreso y Salud" or AQuAS or "Agencia de Qualitat i Avaluacio Sanitaries de Catalunya" or "Agency for Health Quality and Assessment of Catalonia" or "AVALIA T" or ("IACS" and "Spain") or "Health Sciences Institute in Aragon" or "Instituto Aragones de Ciencias de la Salud" or OSTEBA or "Agencia Española de Medicamentos y Productos Sanitarios" or "The Instituto De Salud Carlos III" or "Fundacion Profesor Novoa Santos" or "Basque Foundation for Health Innovation and Research" or "Directorate General for Pharmacy and Health Care Products" or "Fundacion Canaria de Investigacion Sanitaria" or ("Evaluation AND Planning Unit" and "Directorate of the Canary Islands Health Service") or "Swedish Dental and Pharmaceutical Benefits Agency" or ("Medical Products Agency" and Sweden) or ("Dental and Pharmaceutical Benefits Agency" and Sweden) or SFOPH or "Swiss Federal Office of Public Health" or "Swiss Network for HTA" or "Healthcare Improvement Scotland" or "National Institute for Health and Care Excellence" or "National Institute for Health and Clinical Excellence" or ("NICE" and "UNITED KINGDOM") or NIHR or ("National Institute for Health Research" and "UNITED KINGDOM") or "All Wales Therapeutics and Toxicology Centre" or "Healthcare Improvement Scotland" or ("International Centre for Community Driven Research" and ("United Kingdom" or Switzerland or Australia)) or AHRQ or ("Agency for Healthcare Research and Quality" and USA) or "Blue Cross Blue Shield Association" or CMTP or ("Center for Medical Technology Policy" and USA) or ("Institute for Clinical & Economic Review" and USA) or "Kaiser Permanente" or PCORI or "Patient Centered Outcomes Research Institute" or "University of Vermont").af. 161616

5 or/1-4 288149

6 exp guidelines as topic/ and ("attitude of health personnel"/ or guideline adherence/ or practice patterns, physicians'/ or Health Knowledge, Attitudes, Practice/) 27719

7 (guideline or guidance).ti. or (guidelines or national guideline* or (Guideline adj (adher* or impact* or implement* or recommendation*))).ti,ab,kf. 445989

8 *practice guidelines as topic/ and (exp Australia/ or new zealand/ or austria/ or belgium/ or exp baltic states/ or czech republic/ or europe/ or hungary/ or poland/ or slovakia/ or slovenia/ or exp france/ or exp germany/ or exp united kingdom/ or greece/ or ireland/ or exp italy/ or luxembourg/ or netherlands/ or portugal/ or exp "scandinavian and nordic countries"/ or spain/ or switzerland/ or costa rica/ or north america/ or exp canada/ or mexico/ or exp united states/ or chile/ or colombia/ or israel/ or turkey/ or exp japan/ or exp "republic of korea"/) 20952

9 or/6-8 465145

10 primary health care/ or physicians, family/ or general practitioners/ or exp general practice/ or physicians, primary care/ or (general practi* or (family adj2 (doctor* or medicine or physician* or practi*)) or (primary adj2 care) or primary health*).mp. 335233

11 Case Reports/ 2317396

12 Organizational Case Studies/ 12633

13 exp Qualitative Research/ 79466

14 qualitative.mp. 330522

15 action research.mp. 5496

16 Community-Based Participatory Research/ 5515

17 participatory research.mp. 8853

18 case stud*.mp. 134055

19 ethno*.mp. 205582

20 grounded theory.mp. 14850

21 phenomeno*.mp. 277594

22 Narration/ 9960

23 narrative*.mp. 77471

24 biograph*.mp. 186954

25 Autobiography/ 3959

26 Autobiograph*.mp. 10300

27 documentar*.mp. 3533

28 active feedback.mp. 293

29 conversation*.mp. 26874

30 discourse*.mp. 20050

31 thematic.mp. 56630

32 key informant*.mp. 10377

33 Focus Groups/ 35207

34 focus group*.mp. 67488

35 case report*.mp. 2412961

36 Interview/ 30533

37 interview*.mp. 475501

38 Observation/ 5905

39 observ*.mp. 4175766

40 visual data.mp. 760

41 (audio adj record*).mp. 10347

42 Anthropology, Cultural/ 7346

43 experience*.mp. 1318183

44 or/11-43 8578995

45 exp clinical trial/ 961901

46 exp Research Design/ 488166

47 random allocation/ 106905

48 double-blind method/ 174205

49 Single-Blind Method/ 32467

50 Placebos/ 35925

51 Cross-Over Studies/ 54641

52 or/45-51 1284184

53 quantitative.mp. 768187

54 (clinic* adj25 trial*).mp. 1248439

55 random*.mp. 1640500

56 control*.mp. 6223955

57 (latin adj square).mp. 5345

58 placebo*.mp. 258151

59 or/53-58 7809556

60 Comparative Study/ 1912026

61 comparative stud*.mp. 1977448

62 Validation Studies/ 109096

63 validation stud*.mp. 127266

64 evaluation studies/ 261794

65 evaluation stud*.mp. 389951

66 Follow-Up Studies/ 689629

67 (followup or follow-up).mp. 1564169

68 longitudinal.mp. 377935

69 Prospective Studies/ 649907

70 Cross-Over Studies/ 54641

71 cross over.mp. 69344

72 crossover.mp. 72391

73 (prospective* or retrospective*).mp. 2299740

74 volunteer*.mp. 236422

75 or/60-74 5653693

76 singl*.mp. 2122206

77 doubl*.mp. 719700

78 trebl*.mp. 532

79 tripl*.mp. 158043

80 or/76-79 2824047

81 mask*.mp. 106406

82 blind*.mp. 414694

83 81 or 82 513297

84 80 and 83 292059

85 52 or 59 or 75 or 84 11648873

86 Cohort Studies/ 324415

87 Case-Control Studies/ 325879

88 Cross-Sectional Studies/ 455277

89 Health Surveys/ 66644

90 Health Care Surveys/ 33989

91 Risk/ 128046

92 Incidence/ 298093

93 Prevalence/ 338885

94 Mortality/ 49188

95 cohort*.mp. 933131

96 case-control.mp. 378727

97 cross sectional.mp. 632818

98 (health* adj2 survey*).mp. 148506

99 risk.mp. 3128895

100 incidence.mp. 1003316

101 prevalence.mp. 860247

102 mortality.tw. 943169

103 "case series".mp. 100205

104 "time series".mp. 44250

105 ("before and after" or "before after").mp. 323475

106 prognos*.mp. 1060939

107 predict*.mp. 2080352

108 course*.mp. 694854

109 or/86-108 8151458

110 (mixed adj5 method*).mp. 46505

111 multimethod*.mp. 2436

112 (multiple adj5 method*).mp. 34859

113 or/110-112 83156

114 44 or 85 or 109 or 113 19420658

115 114 not (comment or editorial or newspaper article).pt. 19027755

116 115 not (exp animals/ not humans.sh.) 16551031

117 5 and 9 and 10 and 116 1318

118 limit 117 to ((english or french) and yr="2008 -Current") 1127

**Embase (Ovid), 1946-**

1 biomedical technology assessment/ 14552

2 (health technolog* or hta or technology assessment*).af. 71551

3 (AHTA or "ASERNIP S" or "Australian Safety and Efficacy Register of New Interventional Procedures Surgical" or (PBAC and MSAC) or ("Australia Government" and "Department of Health and Ageing") or ("Menzies School of Public" and "University of Sydney") or "The George Institute for Global Health" or AIHTA or GOeG or "Gesunheit Osterreich GmbH" or "Hauptverband der Osterreichischen Sozialversicherungstrager" or "Association of Austrian Social Insurance Institutions" or ("University for Health Sciences" and "Medical Informatics and Technology") or "Belgian Health Care Knowledge Centre" or ("Scientific Institute of Public Health" and "Belgium") or ("Rijksinstituut voor Ziekte" and "en Invaliditeitsverzekering") or CADTH or "Canadian Agency for Drugs and Technologies in Health" or (IHE and CANADA) or ("Institute of Health Economics" and CANADA) or "Institut national d’excellence en sante et en services sociaux" or "National Institute for Excellence in Health and Social Services" or INESSS or ("Ministry of Health" and "Chile") or (("Institute of Public Health" or "ISP") and Chile) or (IETS and Colombia) or "Instituto de Evaluacion Tecnologica en Salud" or ("Ministry of Health and Social Protection" and Colombia) or IETS or "Costa Rican Social Security Fund" or (CCSS and "Costa Rica") or ("Directorate of Scientific and Technological Development in Health" and "Ministry of Health" and "Costa Rica") or "Ministry of Health of the Czech Republic" or ("State Institute for Drug Control" and "Czech Republic") or DEFACTUM or ("Social & Health Services and Labour Market" and "DENMARK") or ("Institute of Family Medicine and Public Health" and "Estonia") or FinCCHTA or "Finnish Medicines Agency" or ("National Institute for Health and Welfare" and Finland) or "Assistance publique Hopitaux de Paris" or "French National Authority for Health" or "Haute Autorite de Sante" or "G BA" or (("The Federal Joint Committee" or "Gemeinsamer Bundesausschuss") and GERMANY) or IQWiG or (("Institut für Qualitat und Wirtschaftlichkeit im Gesundheitswesen" or "Institute for Quality and Efficiency in Health Care") and Germany) or "German Institute for Medical Documentation and Information" or "National and Kapodistrian University of Athens" or "National Evalution Center of Quality and Technology in S.A." or EKAPTY or ("National Organization for Medicines" and Greece) or ("National Organisation for Healthcare Provision" and Greece) or ("Institute of Pharmaceutical Research and Technology" and Greece) or "Onassis Cardiac Surgery Centre" or ("National Institute of Pharmacy and Nutrition" and Hungary) or ("Health Services Management Training Center" and Hungary) or HIQA or ("Health Information and Quality Authority" and IRELAND) or ("National Centre for Pharmacoeconomics" and "St. James Hospital") or Agenas or ("The Agency for Regional Healthcare" and ITALY) or (ASSR and ITALY) or (("Agenzia Sanitaria e Sociale Regionale" or "Regional Agency for Health and Social Care") and ITALY) or "HTA Unit in A. Gemelli Teaching Hospital" or "University Hospital A. Gemelli" or ("Agenzia Sanitaria e Sociale Regionale" and "Regione Emilia Romagna") or ("National Agency for Regional Health Services" and Italy) or "Italian Medicines Agency" or "Centro Regionale Unico sul Farmaca del Veneta" or ("Sede del Ministro" and "Ministero della salute") or "Regione Emilia Romagna" or ("Regione Del Veneto" and "Area Sanita E Sociale")).af. 99693

4 ((("Center for Outcomes Research and Economic Evaluation for Health" or "C2H") and "National Institute of Public Health") or ("Research Center for Health Policy and Economics" and ("Hitotsubashi Institute for Advanced Study" or HIAS)) or ("National Health Service" and Latvia) or ("The Institute of Hygiene" and Lithuania) or ("State Health Care Accreditation Agency" and Lithuania) or "State Medicines Control Agency of Lithuania" or (("National Center for Technological Excellence in Health" or "CENETEC") and "PAHO WHO Collaborating Center") or "Zorginstituut Nederland" or ("ZonMw" and Netherlands) or "The Netherlands Organisation for Health Research and Development" or ZINL or ("National Health Care Institute" and Netherlands) or "Erasmus Universiteit Rotterdam" or "Utrecht University" or "Norwegian Institute of Public Health" or NIPHNO or "Norwegian Institute of Public Health Norway" or "Norwegian Centre for E health Research" or "Norwegian Directorate of Health" or "Norwegian Medicines Agency" or AOTMiT or ("Administracao Central do Sistema de Saude" and "I.P.") or ("National Authority of Medicines and Health Products" and Portugal) or "Ministry of Health of the Slovak Republic" or "Comenius University in Bratislava" or "Public Agency of the Republic of Slovenia for Medicinal Products and Medical Devices" or "Ministry of Health of the Republic of Slovenia" or ("National instute of Public Health" and Slovenia) or NIJZ or ("NECA" and "South Korea") or ("National Evidence based Healthcare Collaborating Agency" and "South Korea") or ("Department of Health Convergence" and "Ewha Womans University") or AETS or "Agencia de Evaluacion de Tecnologias Sanitarias" or AETSA or "Fundacion Publica Andaluza Progreso y Salud" or AQuAS or "Agencia de Qualitat i Avaluacio Sanitaries de Catalunya" or "Agency for Health Quality and Assessment of Catalonia" or "AVALIA T" or ("IACS" and "Spain") or "Health Sciences Institute in Aragon" or "Instituto Aragones de Ciencias de la Salud" or OSTEBA or "Agencia Española de Medicamentos y Productos Sanitarios" or "The Instituto De Salud Carlos III" or "Fundacion Profesor Novoa Santos" or "Basque Foundation for Health Innovation and Research" or "Directorate General for Pharmacy and Health Care Products" or "Fundacion Canaria de Investigacion Sanitaria" or ("Evaluation AND Planning Unit" and "Directorate of the Canary Islands Health Service") or "Swedish Dental and Pharmaceutical Benefits Agency" or ("Medical Products Agency" and Sweden) or ("Dental and Pharmaceutical Benefits Agency" and Sweden) or SFOPH or "Swiss Federal Office of Public Health" or "Swiss Network for HTA" or "Healthcare Improvement Scotland" or "National Institute for Health and Care Excellence" or "National Institute for Health and Clinical Excellence" or ("NICE" and "UNITED KINGDOM") or NIHR or ("National Institute for Health Research" and "UNITED KINGDOM") or "All Wales Therapeutics and Toxicology Centre" or "Healthcare Improvement Scotland" or ("International Centre for Community Driven Research" and ("United Kingdom" or Switzerland or Australia)) or AHRQ or ("Agency for Healthcare Research and Quality" and USA) or "Blue Cross Blue Shield Association" or CMTP or ("Center for Medical Technology Policy" and USA) or ("Institute for Clinical & Economic Review" and USA) or "Kaiser Permanente" or PCORI or "Patient Centered Outcomes Research Institute" or "University of Vermont").af. 286106

5 or/1-4 444001

6 *practice guideline/ and (health personnel attitude/ or physician attitude/ or "alert fatigue (health care)"/ or protocol compliance/) 3936

7 (guideline or guidance).ti. or (guidelines or national guideline* or (Guideline adj (adher* or impact* or implement* or recommendation*))).ti,ab,kf. 657501

8 *practice guideline/ and (exp "australia and new zealand"/ or exp baltic states/ or exp Czechoslovakia/ or hungary/ or exp western europe/ or poland/ or exp southern europe/ or exp scandinavia/ or exp north america/ or costa rica/ or chile/ or colombia/ or israel/ or japan/ or south korea/ or slovenia/ or "turkey (republic)"/) 23935

9 6 or 7 or 8 667071

10 exp *primary health care/ or general practitioner/ or general practice/ or (general practi* or (family adj2 (doctor* or medicine or physician* or practi*)) or (primary adj2 care) or primary health*).mp. 407297

11 health services research/ 29974

12 exp qualitative research/ 109651

13 qualitative.mp. 399403

14 action research.mp. 6457

15 Participatory Research/ 6860

16 participatory research.mp. 9325

17 case stud*.mp. 218064

18 ethno*.mp. 106734

19 grounded theory.mp. 18148

20 phenomeno*.mp. 279963

21 Narrative/ 22737

22 narrative*.mp. 77639

23 biograph*.mp. 14058

24 Autobiograph*.mp. 6925

25 documentar*.mp. 3937

26 active feedback.mp. 285

27 conversation*.mp. 36544

28 discourse*.mp. 20745

29 thematic.mp. 68266

30 key informant*.mp. 11508

31 focus group*.mp. 75576

32 case report*.mp. 2138244

33 exp Interview/ 337993

34 interview*.mp. 560680

35 exp Observational method/ 7270

36 observ*.mp. 4538856

37 visual data.mp. 849

38 (audio adj record*).mp. 16111

39 Cultural Anthropology/ 43775

40 experience*.mp. 1663800

41 or/11-40 8797291

42 exp clinical study/ 9550241

43 exp human experimentation/ 493975

44 exp Methodology/ 6313478

45 randomization/ 89379

46 placebo/ 343071

47 Crossover procedure/ 68829

48 or/42-47 13085915

49 quantitative.mp. 1066516

50 (clinic* adj25 trial*).mp. 1927052

51 random*.mp. 2003809

52 control*.mp. 12067343

53 (latin adj square).mp. 4466

54 placebo*.mp. 434686

55 or/49-54 13642957

56 exp Comparative Study/ 1262555

57 comparative stud*.mp. 811233

58 Validation Study/ 100677

59 validation stud*.mp. 112813

60 evaluation research/ 2116

61 evaluation stud*.mp. 64434

62 Follow-Up/ 1897397

63 (followup or follow up).mp. 2273340

64 longitudinal.mp. 439946

65 cross over.mp. 29266

66 crossover.mp. 98838

67 (prospective* or retrospective*).mp. 3043365

68 volunteer*.mp. 243953

69 or/56-68 6127106

70 singl*.mp. 2501963

71 doubl*.mp. 775967

72 trebl*.mp. 470

73 tripl*.mp. 203768

74 or/70-73 3276922

75 mask*.mp. 135064

76 blind*.mp. 496293

77 75 or 76 622398

78 74 and 77 338101

79 48 or 55 or 69 or 78 20357643

80 exp Health Survey/ 245543

81 Health Care Survey/ 22471

82 exp Risk/ 2854861

83 exp Incidence/ 617442

84 exp Prevalence/ 921420

85 exp Mortality/ 1221855

86 cohort*.mp. 1557044

87 case-control.mp. 270037

88 cross sectional.mp. 741662

89 (health* adj2 survey*).mp. 269149

90 risk.mp. 4465577

91 incidence.mp. 1280707

92 prevalence.mp. 1239948

93 mortality.tw. 1285785

94 "case series".mp. 140948

95 "time series".mp. 60081

96 ("before and after" or before after).mp. 391706

97 prognos*.mp. 1261781

98 predict*.mp. 2598998

99 course*.mp. 1171899

100 or/80-99 10731097

101 (mixed adj5 method*).mp. 55246

102 multimethod*.mp. 2470

103 (multiple adj5 method*).mp. 57452

104 or/101-103 114297

105 41 or 79 or 100 or 104 23553008

106 105 not editorial/ 23294898

107 106 not ((exp animal/ or animal experiment/ or nonhuman/) not (exp human/ or human experiment/)) 19479827

108 5 and 9 and 10 and 107 2984

109 limit 108 to ((english or french) and yr="2008 -Current") 2701

**APA PsycInfo (Ovid), 2002-**

1 (health technolog* or hta or technology assessment*).af. 23799

2 (AHTA or "ASERNIP S" or "Australian Safety and Efficacy Register of New Interventional Procedures Surgical" or (PBAC and MSAC) or ("Australia Government" and "Department of Health and Ageing") or ("Menzies School of Public" and "University of Sydney") or "The George Institute for Global Health" or AIHTA or GOeG or "Gesunheit Osterreich GmbH" or "Hauptverband der Osterreichischen Sozialversicherungstrager" or "Association of Austrian Social Insurance Institutions" or ("University for Health Sciences" and "Medical Informatics and Technology") or "Belgian Health Care Knowledge Centre" or ("Scientific Institute of Public Health" and "Belgium") or ("Rijksinstituut voor Ziekte" and "en Invaliditeitsverzekering") or CADTH or "Canadian Agency for Drugs and Technologies in Health" or (IHE and CANADA) or ("Institute of Health Economics" and CANADA) or "Institut national d’excellence en sante et en services sociaux" or "National Institute for Excellence in Health and Social Services" or INESSS or ("Ministry of Health" and "Chile") or (("Institute of Public Health" or "ISP") and Chile) or (IETS and Colombia) or "Instituto de Evaluacion Tecnologica en Salud" or ("Ministry of Health and Social Protection" and Colombia) or IETS or "Costa Rican Social Security Fund" or (CCSS and "Costa Rica") or ("Directorate of Scientific and Technological Development in Health" and "Ministry of Health" and "Costa Rica") or "Ministry of Health of the Czech Republic" or ("State Institute for Drug Control" and "Czech Republic") or DEFACTUM or ("Social & Health Services and Labour Market" and "DENMARK") or ("Institute of Family Medicine and Public Health" and "Estonia") or FinCCHTA or "Finnish Medicines Agency" or ("National Institute for Health and Welfare" and Finland) or "Assistance publique Hopitaux de Paris" or "French National Authority for Health" or "Haute Autorite de Sante" or "G BA" or (("The Federal Joint Committee" or "Gemeinsamer Bundesausschuss") and GERMANY) or IQWiG or (("Institut für Qualitat und Wirtschaftlichkeit im Gesundheitswesen" or "Institute for Quality and Efficiency in Health Care") and Germany) or "German Institute for Medical Documentation and Information" or "National and Kapodistrian University of Athens" or "National Evalution Center of Quality and Technology in S.A." or EKAPTY or ("National Organization for Medicines" and Greece) or ("National Organisation for Healthcare Provision" and Greece) or ("Institute of Pharmaceutical Research and Technology" and Greece) or "Onassis Cardiac Surgery Centre" or ("National Institute of Pharmacy and Nutrition" and Hungary) or ("Health Services Management Training Center" and Hungary) or HIQA or ("Health Information and Quality Authority" and IRELAND) or ("National Centre for Pharmacoeconomics" and "St. James Hospital") or Agenas or ("The Agency for Regional Healthcare" and ITALY) or (ASSR and ITALY) or (("Agenzia Sanitaria e Sociale Regionale" or "Regional Agency for Health and Social Care") and ITALY) or "HTA Unit in A. Gemelli Teaching Hospital" or "University Hospital A. Gemelli" or ("Agenzia Sanitaria e Sociale Regionale" and "Regione Emilia Romagna") or ("National Agency for Regional Health Services" and Italy) or "Italian Medicines Agency" or "Centro Regionale Unico sul Farmaca del Veneta" or ("Sede del Ministro" and "Ministero della salute") or "Regione Emilia Romagna" or ("Regione Del Veneto" and "Area Sanita E Sociale")).af. 8506

3 ((("Center for Outcomes Research and Economic Evaluation for Health" or "C2H") and "National Institute of Public Health") or ("Research Center for Health Policy and Economics" and ("Hitotsubashi Institute for Advanced Study" or HIAS)) or ("National Health Service" and Latvia) or ("The Institute of Hygiene" and Lithuania) or ("State Health Care Accreditation Agency" and Lithuania) or "State Medicines Control Agency of Lithuania" or (("National Center for Technological Excellence in Health" or "CENETEC") and "PAHO WHO Collaborating Center") or "Zorginstituut Nederland" or ("ZonMw" and Netherlands) or "The Netherlands Organisation for Health Research and Development" or ZINL or ("National Health Care Institute" and Netherlands) or "Erasmus Universiteit Rotterdam" or "Utrecht University" or "Norwegian Institute of Public Health" or NIPHNO or "Norwegian Institute of Public Health Norway" or "Norwegian Centre for E health Research" or "Norwegian Directorate of Health" or "Norwegian Medicines Agency" or AOTMiT or ("Administracao Central do Sistema de Saude" and "I.P.") or ("National Authority of Medicines and Health Products" and Portugal) or "Ministry of Health of the Slovak Republic" or "Comenius University in Bratislava" or "Public Agency of the Republic of Slovenia for Medicinal Products and Medical Devices" or "Ministry of Health of the Republic of Slovenia" or ("National instute of Public Health" and Slovenia) or NIJZ or ("NECA" and "South Korea") or ("National Evidence based Healthcare Collaborating Agency" and "South Korea") or ("Department of Health Convergence" and "Ewha Womans University") or AETS or "Agencia de Evaluacion de Tecnologias Sanitarias" or AETSA or "Fundacion Publica Andaluza Progreso y Salud" or AQuAS or "Agencia de Qualitat i Avaluacio Sanitaries de Catalunya" or "Agency for Health Quality and Assessment of Catalonia" or "AVALIA T" or ("IACS" and "Spain") or "Health Sciences Institute in Aragon" or "Instituto Aragones de Ciencias de la Salud" or OSTEBA or "Agencia Española de Medicamentos y Productos Sanitarios" or "The Instituto De Salud Carlos III" or "Fundacion Profesor Novoa Santos" or "Basque Foundation for Health Innovation and Research" or "Directorate General for Pharmacy and Health Care Products" or "Fundacion Canaria de Investigacion Sanitaria" or ("Evaluation AND Planning Unit" and "Directorate of the Canary Islands Health Service") or "Swedish Dental and Pharmaceutical Benefits Agency" or ("Medical Products Agency" and Sweden) or ("Dental and Pharmaceutical Benefits Agency" and Sweden) or SFOPH or "Swiss Federal Office of Public Health" or "Swiss Network for HTA" or "Healthcare Improvement Scotland" or "National Institute for Health and Care Excellence" or "National Institute for Health and Clinical Excellence" or ("NICE" and "UNITED KINGDOM") or NIHR or ("National Institute for Health Research" and "UNITED KINGDOM") or "All Wales Therapeutics and Toxicology Centre" or "Healthcare Improvement Scotland" or ("International Centre for Community Driven Research" and ("United Kingdom" or Switzerland or Australia)) or AHRQ or ("Agency for Healthcare Research and Quality" and USA) or "Blue Cross Blue Shield Association" or CMTP or ("Center for Medical Technology Policy" and USA) or ("Institute for Clinical & Economic Review" and USA) or "Kaiser Permanente" or PCORI or "Patient Centered Outcomes Research Institute" or "University of Vermont").af. 80634

4 or/1-3 108749

5 treatment guidelines/ and (exp health personnel attitudes/ or Health Knowledge/) 189

6 (guideline or guidance).ti. or (guidelines or national guideline* or (Guideline adj (adher* or impact* or implement* or recommendation*))).mp. 65968

7 treatment guidelines/ and (australia or austria or baltic state* or belgium or canada or chile or colombia or costa rica or czech or denmark or estonia or europ* or finland or france or germany or greece or hungary or iceland or ireland or israel or italy or japan or korea or latvia or lithuania or luxembourg or mexico or netherlands or new zealand or north america* or norway or poland or portugal or scandinavia* or slovak* or slovenia or spain or switzerland or turkey or united states or usa or uk or united kingdom).mp. 2138

8 5 or 6 or 7 65968

9 primary health care/ or family medicine/ or family physicians/ or general practitioners/ or (general practi* or (family adj2 (doctor* or medicine or physician* or practi*)) or (primary adj2 care) or primary health*).mp. 53830

10 Case Report/ 1363

11 qualitative.mp. 196321

12 action research.mp. 10294

13 participatory research.mp. 4493

14 case stud*.mp. 90238

15 ethno*.mp. 35584

16 grounded theory.mp. 17502

17 phenomeno*.mp. 101205

18 narrative*.mp. 72799

19 biograph*.mp. 9579

20 exp Biography/ 3093

21 Autobiograph*.mp. 10538

22 documentar*.mp. 3075

23 active feedback.mp. 37

24 conversation*.mp. 35580

25 discourse*.mp. 54140

26 thematic.mp. 36750

27 key informant*.mp. 3755

28 focus group*.mp. 43120

29 case report*.mp. 22179

30 exp Interviews/ 12609

31 interview*.mp. 385632

32 exp Observation Methods/ 3605

33 observ*.mp. 383825

34 visual data.mp. 415

35 (audio adj record*).mp. 5624

36 "Culture (Anthropological)"/ 14564

37 experience*.mp. 596807

38 or/10-37 1389815

39 exp Quantitative Methods/ 3595

40 quantitative.mp. 101370

41 exp Empirical Methods/ 35944

42 exp Experimentation/ 208346

43 Placebo/ 5150

44 or/39-43 302016

45 (clinic* adj25 trial*).mp. 54831

46 random*.mp. 198481

47 control*.mp. 602096

48 (latin adj square).mp. 225

49 placebo*.mp. 30180

50 or/45-49 720460

51 comparative stud*.mp. 9622

52 exp Test Validity/ 68725

53 validation stud*.mp. 4595

54 Program Evaluation/ 8956

55 evaluation stud*.mp. 3739

56 (followup or follow up).mp. 121015

57 longitudinal.mp. 127730

58 cross over.mp. 6117

59 crossover.mp. 6513

60 (prospective* or retrospective*).mp. 124697

61 volunteer*.mp. 31650

62 or/51-61 435562

63 singl*.mp. 161979

64 doubl*.mp. 46235

65 trebl*.mp. 49

66 tripl*.mp. 5904

67 or/63-66 207715

68 mask*.mp. 15194

69 blind*.mp. 45121

70 68 or 69 59642

71 67 and 70 27998

72 44 or 50 or 62 or 71 1229396

73 Risk Factors/ 93081

74 observational.mp. 28886

75 cohort*.mp. 96382

76 case-control.mp. 32368

77 cross sectional.mp. 117523

78 (health* adj2 survey*).mp. 47065

79 risk.mp. 406775

80 incidence.mp. 41924

81 prevalence.mp. 122812

82 mortality.tw. 41450

83 "case series".mp. 4477

84 "time series".mp. 8065

85 ("before and after" or before after).mp. 35890

86 prognos*.mp. 27486

87 predict*.mp. 402098

88 course*.mp. 151208

89 or/73-88 1118608

90 (mixed adj5 method*).mp. 35405

91 multimethod*.mp. 2530

92 (multiple adj5 method*).mp. 9645

93 or/90-92 46917

94 38 or 72 or 89 or 93 2444489

95 94 not (comment or editorial).dt. 2424042

96 4 and 8 and 9 and 95 532

97 limit 96 to ((english or french) and yr="2008 -Current") 454

**CINAHL Plus with Full Text (EBSCOhost), Inception-**

| **#** | **Query** | **Limiters/Expanders** | **Last Run Via** | **Results** |
| --- | --- | --- | --- | --- |
| S15 | PY 2008- AND LA ( (english OR french) ) AND S14 | Expanders - Apply equivalent subjects Search modes - Boolean/Phrase | Interface - EBSCOhost Research Databases Search Screen - Advanced Search Database - CINAHL Plus with Full Text | 1,939 |
| S14 | S6 AND S9 AND S10 AND S13 | Expanders - Apply equivalent subjects Search modes - Boolean/Phrase | Interface - EBSCOhost Research Databases Search Screen - Advanced Search Database - CINAHL Plus with Full Text | 2,268 |
| S13 | S11 OR S12 | Expanders - Apply equivalent subjects Search modes - Boolean/Phrase | Interface - EBSCOhost Research Databases Search Screen - Advanced Search Database - CINAHL Plus with Full Text | 4,672,693 |
| S12 | (qualitative OR "action research" OR "participatory research" OR "case stud*" OR ethno* OR "grounded theory" OR phenomeno* OR narrat* OR biograph* OR autobiograph* OR documentar* OR "active feedback" OR conversation* OR discourse* OR thematic OR "key informant*" OR "focus group*" OR "case report*" OR interview* OR observ* OR "visual data" OR (audio N1 record*) OR experience* OR quantitative OR (clinic* N25 trial*) OR random* OR control* OR (latin N1 square) OR placebo* OR "comparative stud*" OR "validation stud*" OR "evaluation stud*" OR followup OR "follow-up" OR longitudinal OR "cross over" OR crossover OR prospective* OR retrospective* OR volunteer* OR ((singl* OR doubl* OR trebl* OR tripl*) AND (mask* OR blind*)) OR cohort* OR case-control OR "cross sectional" OR (health* N2 survey*) OR risk OR incidence OR prevalence OR mortality OR "case series" OR "time series" OR "before and after" OR "before after" OR prognos* OR predict* OR course* OR (mixed N5 method*) OR multimethod* OR (multiple N5 method*)) | Expanders - Apply equivalent subjects Search modes - Boolean/Phrase | Interface - EBSCOhost Research Databases Search Screen - Advanced Search Database - CINAHL Plus with Full Text | 4,384,149 |
| S11 | (MH "Research by Type and Subject+") OR (MH "Research Methodology+") | Expanders - Apply equivalent subjects Search modes - Boolean/Phrase | Interface - EBSCOhost Research Databases Search Screen - Advanced Search Database - CINAHL Plus with Full Text | 2,715,969 |
| S10 | (MH "Primary Health Care") OR (MH "Physicians, Family") OR (MH "Family Practice") OR (general practi* or (family N2 (doctor* or medicine or physician* or practi*)) or (primary N2 care) or primary health*) | Expanders - Apply equivalent subjects Search modes - Boolean/Phrase | Interface - EBSCOhost Research Databases Search Screen - Advanced Search Database - CINAHL Plus with Full Text | 187,055 |
| S9 | S7 OR S8 | Expanders - Apply equivalent subjects Search modes - Boolean/Phrase | Interface - EBSCOhost Research Databases Search Screen - Advanced Search Database - CINAHL Plus with Full Text | 551,647 |
| S8 | TI(guideline or guidance) or (guidelines or national guideline* or (Guideline N1 (adher* or impact* or implement* or recommendation*))) | Expanders - Apply equivalent subjects Search modes - Boolean/Phrase | Interface - EBSCOhost Research Databases Search Screen - Advanced Search Database - CINAHL Plus with Full Text | 242,834 |
| S7 | (MH "Practice Guidelines") AND (MH "Attitude of Health Personnel") OR (MH "Physician Attitudes" OR MH "Guideline Adherence" OR MH "Professional Practice+" OR ( MM "Practice Guidelines" AND ((MH "Australia+") OR (MH "New Zealand") OR (MH "Austria") OR (MH "Belgium") OR (MH "Canada+") OR (MH "Chile") OR (MH "North America") OR (MH "Colombia") OR (MH "Costa Rica") OR (MH "Czech Republic") OR (MH "France") OR (MH "Germany+") OR (MH "Greece") OR (MH "Iceland") OR (MH "Ireland") OR (MH "Hungary") OR (MH "Italy") OR (MH "Japan") OR (MH "South Korea") OR (MH "Israel") OR (MH "Baltic States+") OR (MH "Luxembourg") OR (MH "Mexico") OR (MH "United States+") OR (MH "Netherlands") OR (MH "Scandinavia") OR (MH "Poland") OR (MH "Portugal") OR (MH "Slovakia") OR (MH "Slovenia") OR (MH "Spain") OR (MH "Switzerland") OR (MH "United Kingdom+") OR (MH "Turkey")))) | Expanders - Apply equivalent subjects Search modes - Boolean/Phrase | Interface - EBSCOhost Research Databases Search Screen - Advanced Search Database - CINAHL Plus with Full Text | 360,652 |
| S6 | S1 OR S2 OR S3 OR S4 OR S5 | Expanders - Apply equivalent subjects Search modes - Boolean/Phrase | Interface - EBSCOhost Research Databases Search Screen - Advanced Search Database - CINAHL Plus with Full Text | 172,521 |
| S5 | TX("Fundacion Publica Andaluza Progreso y Salud" or AQuAS or "Agencia de Qualitat i Avaluacio Sanitaries de Catalunya" or "Agency for Health Quality and Assessment of Catalonia" or "AVALIA T" or ("IACS" and "Spain") or "Health Sciences Institute in Aragon" or "Instituto Aragones de Ciencias de la Salud" or OSTEBA or "Agencia Española de Medicamentos y Productos Sanitarios" or "The Instituto De Salud Carlos III" or "Fundacion Profesor Novoa Santos" or "Basque Foundation for Health Innovation and Research" or "Directorate General for Pharmacy and Health Care Products" or "Fundacion Canaria de Investigacion Sanitaria" or ("Evaluation AND Planning Unit" and "Directorate of the Canary Islands Health Service") or "Swedish Dental and Pharmaceutical Benefits Agency" or ("Medical Products Agency" and Sweden) or ("Dental and Pharmaceutical Benefits Agency" and Sweden) or SFOPH or "Swiss Federal Office of Public Health" or "Swiss Network for HTA" or "Healthcare Improvement Scotland" or "National Institute for Health and Care Excellence" or "National Institute for Health and Clinical Excellence" or ("NICE" and "UNITED KINGDOM") or NIHR or ("National Institute for Health Research" and "UNITED KINGDOM") or "All Wales Therapeutics and Toxicology Centre" or "Healthcare Improvement Scotland" or ("International Centre for Community Driven Research" and ("United Kingdom" or Switzerland or Australia)) or AHRQ or ("Agency for Healthcare Research and Quality" and USA) or "Blue Cross Blue Shield Association" or CMTP or ("Center for Medical Technology Policy" and USA) or ("Institute for Clinical & Economic Review" and USA) or "Kaiser Permanente" or PCORI or "Patient Centered Outcomes Research Institute" or "University of Vermont") | Expanders - Apply equivalent subjects Search modes - Boolean/Phrase | Interface - EBSCOhost Research Databases Search Screen - Advanced Search Database - CINAHL Plus with Full Text | 101,287 |
| S4 | TX((("Center for Outcomes Research and Economic Evaluation for Health" or "C2H") and "National Institute of Public Health") or ("Research Center for Health Policy and Economics" and ("Hitotsubashi Institute for Advanced Study" or HIAS)) or ("National Health Service" and Latvia) or ("The Institute of Hygiene" and Lithuania) or ("State Health Care Accreditation Agency" and Lithuania) or "State Medicines Control Agency of Lithuania" or (("National Center for Technological Excellence in Health" or "CENETEC") and "PAHO WHO Collaborating Center") or "Zorginstituut Nederland" or ("ZonMw" and Netherlands) or "The Netherlands Organisation for Health Research and Development" or ZINL or ("National Health Care Institute" and Netherlands) or "Erasmus Universiteit Rotterdam" or "Utrecht University" or "Norwegian Institute of Public Health" or NIPHNO or "Norwegian Institute of Public Health Norway" or "Norwegian Centre for E health Research" or "Norwegian Directorate of Health" or "Norwegian Medicines Agency" or AOTMiT or ("Administracao Central do Sistema de Saude" and "I.P.") or ("National Authority of Medicines and Health Products" and Portugal) or "Ministry of Health of the Slovak Republic" or "Comenius University in Bratislava" or "Public Agency of the Republic of Slovenia for Medicinal Products and Medical Devices" or "Ministry of Health of the Republic of Slovenia" or ("National instute of Public Health" and Slovenia) or NIJZ or ("NECA" and "South Korea") or ("National Evidence based Healthcare Collaborating Agency" and "South Korea") or ("Department of Health Convergence" and "Ewha Womans University") or AETS or "Agencia de Evaluacion de Tecnologias Sanitarias" or AETSA) | Expanders - Apply equivalent subjects Search modes - Boolean/Phrase | Interface - EBSCOhost Research Databases Search Screen - Advanced Search Database - CINAHL Plus with Full Text | 16,077 |
| S3 | TX(("National Institute for Health and Welfare" and Finland) or "Assistance publique Hopitaux de Paris" or "French National Authority for Health" or "Haute Autorite de Sante" or "G BA" or (("The Federal Joint Committee" or "Gemeinsamer Bundesausschuss") and GERMANY) or IQWiG or (("Institut für Qualitat und Wirtschaftlichkeit im Gesundheitswesen" or "Institute for Quality and Efficiency in Health Care") and Germany) or "German Institute for Medical Documentation and Information" or "National and Kapodistrian University of Athens" or "National Evalution Center of Quality and Technology in S.A." or EKAPTY or ("National Organization for Medicines" and Greece) or ("National Organisation for Healthcare Provision" and Greece) or ("Institute of Pharmaceutical Research and Technology" and Greece) or "Onassis Cardiac Surgery Centre" or ("National Institute of Pharmacy and Nutrition" and Hungary) or ("Health Services Management Training Center" and Hungary) or HIQA or ("Health Information and Quality Authority" and IRELAND) or ("National Centre for Pharmacoeconomics" and "St. James Hospital") or Agenas or ("The Agency for Regional Healthcare" and ITALY) or (ASSR and ITALY) or (("Agenzia Sanitaria e Sociale Regionale" or "Regional Agency for Health and Social Care") and ITALY) or "HTA Unit in A. Gemelli Teaching Hospital" or "University Hospital A. Gemelli" or ("Agenzia Sanitaria e Sociale Regionale" and "Regione Emilia Romagna") or ("National Agency for Regional Health Services" and Italy) or "Italian Medicines Agency" or "Centro Regionale Unico sul Farmaca del Veneta" or ("Sede del Ministro" and "Ministero della salute") or "Regione Emilia Romagna" or ("Regione Del Veneto" and "Area Sanita E Sociale")) | Expanders - Apply equivalent subjects Search modes - Boolean/Phrase | Interface - EBSCOhost Research Databases Search Screen - Advanced Search Database - CINAHL Plus with Full Text | 12,081 |
| S2 | TX(AHTA or "ASERNIP S" or "Australian Safety and Efficacy Register of New Interventional Procedures Surgical" or (PBAC and MSAC) or ("Australia Government" and "Department of Health and Ageing") or ("Menzies School of Public" and "University of Sydney") or "The George Institute for Global Health" or AIHTA or GOeG or "Gesunheit Osterreich GmbH" or "Hauptverband der Osterreichischen Sozialversicherungstrager" or "Association of Austrian Social Insurance Institutions" or ("University for Health Sciences" and "Medical Informatics and Technology") or "Belgian Health Care Knowledge Centre" or ("Scientific Institute of Public Health" and "Belgium") or ("Rijksinstituut voor Ziekte" and "en Invaliditeitsverzekering") or CADTH or "Canadian Agency for Drugs and Technologies in Health" or (IHE and CANADA) or ("Institute of Health Economics" and CANADA) or "Institut national d’excellence en sante et en services sociaux" or "National Institute for Excellence in Health and Social Services" or INESSS or ("Ministry of Health" and "Chile") or (("Institute of Public Health" or "ISP") and Chile) or (IETS and Colombia) or "Instituto de Evaluacion Tecnologica en Salud" or ("Ministry of Health and Social Protection" and Colombia) or IETS or "Costa Rican Social Security Fund" or (CCSS and "Costa Rica") or ("Directorate of Scientific and Technological Development in Health" and "Ministry of Health" and "Costa Rica") or "Ministry of Health of the Czech Republic" or ("State Institute for Drug Control" and "Czech Republic") or DEFACTUM or ("Social & Health Services and Labour Market" and "DENMARK") or ("Institute of Family Medicine and Public Health" and "Estonia") or FinCCHTA or "Finnish Medicines Agency") | Expanders - Apply equivalent subjects Search modes - Boolean/Phrase | Interface - EBSCOhost Research Databases Search Screen - Advanced Search Database - CINAHL Plus with Full Text | 9,790 |
| S1 | TX(health technolog* or hta or technology assessment*) | Expanders - Apply equivalent subjects Search modes - Boolean/Phrase | Interface - EBSCOhost Research Databases Search Screen - Advanced Search Database - CINAHL Plus with Full Text | 40,721 |

**CENTRAL (Cochrane Library)**

Search Name:

Date Run: 06/02/2023 20:35:56

Comment:

ID Search Hits

#1 (health NEXT technolog* or hta or technology NEXT assessment*) 4793

#2 (AHTA or "ASERNIP S" or "Australian Safety and Efficacy Register of New Interventional Procedures Surgical" or (PBAC and MSAC) or ("Australia Government" and "Department of Health and Ageing") or ("Menzies School of Public" and "University of Sydney") or "The George Institute for Global Health" or AIHTA or GOeG or "Gesunheit Osterreich GmbH" or "Hauptverband der Osterreichischen Sozialversicherungstrager" or "Association of Austrian Social Insurance Institutions" or ("University for Health Sciences" and "Medical Informatics and Technology") or "Belgian Health Care Knowledge Centre" or ("Scientific Institute of Public Health" and "Belgium") or ("Rijksinstituut voor Ziekte" and "en Invaliditeitsverzekering") or CADTH or "Canadian Agency for Drugs and Technologies in Health" or (IHE and CANADA) or ("Institute of Health Economics" and CANADA) or "Institut national d’excellence en sante et en services sociaux" or "National Institute for Excellence in Health and Social Services" or INESSS or ("Ministry of Health" and "Chile") or (("Institute of Public Health" or "ISP") and Chile) or (IETS and Colombia) or "Instituto de Evaluacion Tecnologica en Salud" or ("Ministry of Health and Social Protection" and Colombia) or IETS or "Costa Rican Social Security Fund" or (CCSS and "Costa Rica") or ("Directorate of Scientific and Technological Development in Health" and "Ministry of Health" and "Costa Rica") or "Ministry of Health of the Czech Republic" or ("State Institute for Drug Control" and "Czech Republic") or DEFACTUM or ("Social & Health Services and Labour Market" and "DENMARK") or ("Institute of Family Medicine and Public Health" and "Estonia") or FinCCHTA or "Finnish Medicines Agency") 528

#3 (("National Institute for Health and Welfare" and Finland) or "Assistance publique Hopitaux de Paris" or "French National Authority for Health" or "Haute Autorite de Sante" or "G BA" or (("The Federal Joint Committee" or "Gemeinsamer Bundesausschuss") and GERMANY) or IQWiG or (("Institut für Qualitat und Wirtschaftlichkeit im Gesundheitswesen" or "Institute for Quality and Efficiency in Health Care") and Germany) or "German Institute for Medical Documentation and Information" or "National and Kapodistrian University of Athens" or "National Evalution Center of Quality and Technology in S.A." or EKAPTY or ("National Organization for Medicines" and Greece) or ("National Organisation for Healthcare Provision" and Greece) or ("Institute of Pharmaceutical Research and Technology" and Greece) or "Onassis Cardiac Surgery Centre" or ("National Institute of Pharmacy and Nutrition" and Hungary) or ("Health Services Management Training Center" and Hungary) or HIQA or ("Health Information and Quality Authority" and IRELAND) or ("National Centre for Pharmacoeconomics" and "St. James Hospital") or Agenas or ("The Agency for Regional Healthcare" and ITALY) or (ASSR and ITALY) or (("Agenzia Sanitaria e Sociale Regionale" or "Regional Agency for Health and Social Care") and ITALY) or "HTA Unit in A. Gemelli Teaching Hospital" or "University Hospital A. Gemelli" or ("Agenzia Sanitaria e Sociale Regionale" and "Regione Emilia Romagna") or ("National Agency for Regional Health Services" and Italy) or "Italian Medicines Agency" or "Centro Regionale Unico sul Farmaca del Veneta" or ("Sede del Ministro" and "Ministero della salute") or "Regione Emilia Romagna" or ("Regione Del Veneto" and "Area Sanita E Sociale")) 1769

#4 ("Fundacion Publica Andaluza Progreso y Salud" or AQuAS or "Agencia de Qualitat i Avaluacio Sanitaries de Catalunya" or "Agency for Health Quality and Assessment of Catalonia" or "AVALIA T" or ("IACS" and "Spain") or "Health Sciences Institute in Aragon" or "Instituto Aragones de Ciencias de la Salud" or OSTEBA or "Agencia Española de Medicamentos y Productos Sanitarios" or "The Instituto De Salud Carlos III" or "Fundacion Profesor Novoa Santos" or "Basque Foundation for Health Innovation and Research" or "Directorate General for Pharmacy and Health Care Products" or "Fundacion Canaria de Investigacion Sanitaria" or ("Evaluation AND Planning Unit" and "Directorate of the Canary Islands Health Service") or "Swedish Dental and Pharmaceutical Benefits Agency" or ("Medical Products Agency" and Sweden) or ("Dental and Pharmaceutical Benefits Agency" and Sweden) or SFOPH or "Swiss Federal Office of Public Health" or "Swiss Network for HTA" or "Healthcare Improvement Scotland" or "National Institute for Health and Care Excellence" or "National Institute for Health and Clinical Excellence" or ("NICE" and "UNITED KINGDOM") or NIHR or ("National Institute for Health Research" and "UNITED KINGDOM") or "All Wales Therapeutics and Toxicology Centre" or "Healthcare Improvement Scotland" or ("International Centre for Community Driven Research" and ("United Kingdom" or Switzerland or Australia)) or AHRQ or ("Agency for Healthcare Research and Quality" and USA) or "Blue Cross Blue Shield Association" or CMTP or ("Center for Medical Technology Policy" and USA) or ("Institute for Clinical & Economic Review" and USA) or "Kaiser Permanente" or PCORI or "Patient Centered Outcomes Research Institute" or "University of Vermont") 9040

#5 #1 OR #2 OR #3 OR #4 14276

#6 (guideline* OR guidance):ti,ab,kw 63842

#7 (general next practi* or (family NEAR/2 (doctor* or medicine* or physician* or practi*)) or (primary NEAR/2 care) or primary health*):ti,ab,kw 165412

#8 #5 AND #6 AND #7 with Publication Year from 2008 to 2023, in Trials 498

**Reference**

1. El Sherif, R., Pluye, P., Gore, G., Granikov, V., & Hong, Q. N. (2016). Performance of a mixed filter to identify relevant studies for mixed studies reviews. Journal of the Medical Library Association: JMLA, 104(1), 47-51. https://doi.org/10.3163/1536-5050.104.1.007
