# Supplementary material for: Outcomes of guidelines from health technology assessment organizations in community-based primary care: a systematic mixed studies review
Source: Int J Technol Assess Health Care. 2024 Nov 14;40(1):e56. doi: 10.1017/S0266462324000370 (PMC11579698; doi:10.1017/S0266462324000370)
Supplement: Baradaran et al. supplementary material [file S0266462324000370sup001.zip › Appendix 3.docx]

| **Appendix 3.** Themes, sub-themes, and their definition. | | |
| --- | --- | --- |
| **Dimension (theme)** | **Type of outcome (sub-theme)** | **Definition** |
| 1. RELEVANCE | 1.1. Relevance | Situational relevance of the guideline according to FPs or patients (depending on the context). |
| 2. COGNITIVE or AFFECTIVE IMPACT | 2.1. Understanding | Understanding the guideline-based information (e.g., clarity, confusion, language barrier) |
|  | 2.2. Learning something new | Guidelines changing or adding to the FP or patient knowledge (awareness) or perspective about something. |
|  | 2.3. Validation | Validating the FP or patient knowledge about some guideline-based information. |
|  | 2.4. Reassurance (trust) | FPs’ or patients’ trusting or doubting (being afraid of) the guidelines. |
|  | 2.5. Remembering | Guidelines helping FPs and patients remember their existing knowledge. |
|  | 2.6. Motivation | Being motivated to follow and adhere to the latest guidelines (e.g., information overload might cause a reduction in motivation). |
|  | 2.7. Satisfaction or dissatisfaction | Satisfaction or dissatisfaction of FPs or patients with the content of the guidelines. |
|  | 2.8. Mis-presentation | FPs or patients believing that a guideline is giving misinformation or is misrepresenting some information. |
|  | 2.9. Disagreement | FPs or patients disagreeing with the content of a guideline. |
|  | 2.10. Guideline potentially harmful | FPs or patients believing that a recommendation in a guideline might cause harm. |
|  | 2.11. Willingness to discuss sensitive information | Guidelines affecting FP willingness to discuss sensitive topics with their patients (usually fearing discomfort for themselves or their patients). |
| 3. USE FOR PRACTICE or PATIENT | 3.1. Conceptual use | Changing thinking around a specific issue regarding a particular patient using guideline-based information. |
|  | 3.2. Legitimating use | Justifying or maintaining an action plan for a patient using guidelines. |
|  | 3.3. Instrumental use | Directly modifying the management plan of a patient with guidelines. |
|  | 3.4. Symbolic use | Discussing guidelines with others, e.g., persuading others to change their course of action. |
| 4. INDIVIDUAL PATIENT HEALTH OUTCOMES | 4.1. Health improvement or harm | Guideline-based information improving the health status of a patient or causing harm to a patient. |
|  | 4.2. Increase or decrease worries | Guideline-based information increasing or decreasing worries among patients. |
|  | 4.3. Preventive care | Guidelines preventing conditions among patients, e.g., risk reduction. |
| 5. ORGANIZATIONAL HEALTH OUTCOMES | 5.1. Clinician-patient relationship | Guidelines improving (or facilitating) or harming the relationship (or interaction) between the patient and their FP. |
|  | 5.2. Referrals | Guidelines improving or worsening the referral process. |
|  | 5.3. Integrated care | Guidelines affecting care transitions or continuity of care for patients. |
|  | 5.4. Standardized care | Guidelines maintaining a standard quality of care for the patient. |
|  | 5.5. Quality of investigations | Guidelines affecting the quality of investigation, e.g., laboratory tests or imaging. |
|  | 5.6. Quality of Diagnosis | Guidelines affecting the quality of diagnosis made by FPs. |
|  | 5.7. Quality of Prescriptions | Guidelines affecting the quality of FP prescribing. |
|  | 5.8. Quality of management of chronic disease | Guidelines affecting the quality of management of a chronic disease, e.g., monitoring. |
|  | 5.9. Healthcare use (efficiency) | Guidelines affecting healthcare use and efficiency, e.g., decreasing or increasing time and resources. |
|  | 5.10. Psychosocial effects | Guidelines and social environment affecting FP thoughts and behaviours, e.g., FPs age or location/type of practice affecting their guideline adherence. |
