# Supplementary material for: Outcomes of guidelines from health technology assessment organizations in community-based primary care: a systematic mixed studies review
Source: Int J Technol Assess Health Care. 2024 Nov 14;40(1):e56. doi: 10.1017/S0266462324000370 (PMC11579698; doi:10.1017/S0266462324000370)
Supplement: Baradaran et al. supplementary material [file S0266462324000370sup001.zip › Appendix 5.docx]

| **Appendix 5. Search results** | |  |  |  |  |
| --- | --- | --- | --- | --- | --- |
| Platform | Database(s) | Database coverage dates | Number of Results | Search Date | Remarks |
| OvidSP | Ovid MEDLINE ALL(R) | 1946 - | 1127 | 2022-02-06 | *Limited to 2008-, English or French* |
| OvidSP | EMBASE | 1996 - | 2701 | 2022-02-06 | *Limited to 2008-, English or French* |
| OvidSP | APA PsycInfo | 2002 - | 454 | 2022-02-06 | *Limited to 2008-, English or French* |
| EBSCOhost | CINAHL | 1937 - | 1939 |  | *Limited to 2008-, English or French* |
| Cochrane Library | CENTRAL (Trials) | Inception - | 498 | 2022-02-06 | *Limited to 2008-* |
|  | **TOTAL NUMBER OF RECORDS** |  | 6719 |  |  |
