# Supplementary material for: Outcomes of guidelines from health technology assessment organizations in community-based primary care: a systematic mixed studies review
Source: Int J Technol Assess Health Care. 2024 Nov 14;40(1):e56. doi: 10.1017/S0266462324000370 (PMC11579698; doi:10.1017/S0266462324000370)
Supplement: Baradaran et al. supplementary material [file S0266462324000370sup001.zip › Appendix 6.docx]

| **Appendix 6.** Study characteristics and MMAT scores. | | | | |
| --- | --- | --- | --- | --- |
| Paper | Year | Country | Design | MMAT score |
| Abdelhamid et al. (1) | 2014 | UK | Mixed methods (quantitative descriptive + qualitative) | 80 |
| Alfirevic et al. (2) | 2020 | UK | Quantitative non-randomized | 100 |
| Andre et al. (3) | 2016 | Sweden | Qualitative studies | 100 |
| Arjunan et al. (4) | 2019 | UK | Quantitative non-randomized | 80 |
| Bakhai et al. (5) | 2010 | UK | Quantitative non-randomized | 80 |
| Bakker et al. (6) | 2017 | Netherlands | Quantitative descriptive | 80 |
| Barrera et al. (7) | 2014 | UK | Quantitative non-randomized | 100 |
| Baughan et al. (8) | 2011 | UK | Quantitative non-randomized | 80 |
| Bedson et al. (9) | 2013 | UK | Quantitative non-randomized | 80 |
| Bhachu et al. (10) | 2021 | UK | Quantitative non-randomized | 80 |
| Bishop et al. (11) | 2015 | UK | Qualitative studies | 100 |
| Blanker et al. (12) | 2012 | Netherlands | Quantitative descriptive | 100 |
| Boivin et al. (13) | 2011 | France | Quantitative descriptive | 100 |
| Bottle et al. (14) | 2018 | UK | Quantitative non-randomized | 100 |
| Brown et al. (15) | 2020 | UK | Quantitative non-randomized | 80 |
| Butalid et al. (16) | 2015 | Netherlands | Quantitative non-randomized | 80 |
| Chen et al. (17) | 2018 | UK | Quantitative non-randomized | 60 |
| Coulthard et al. (18) | 2014 | UK | Quantitative non-randomized | 100 |
| Cragg et al. (19) | 2021 | UK | Quantitative non-randomized | 80 |
| Crawford et al. (20) | 2018 | UK | Quantitative non-randomized | 80 |
| Cubiella et al. (21) | 2014 | UK | Quantitative non-randomized | 100 |
| Curtis et al. (22) | 2018 | UK | Quantitative non-randomized | 80 |
| Cuvellier et al. (23) | 2009 | France | Quantitative descriptive | 60 |
| Czarnawska-Iliev et al. (24) | 2016 | UK | Mixed methods (quantitative descriptive + qualitative) | 80 |
| Damery et al. (25) | 2011 | UK | Quantitative non-randomized | 60 |
| Davies et al. (26) | 2018 | UK | Quantitative non-randomized | 80 |
| Davies et al. (27) | 2017 | UK | Quantitative non-randomized | 80 |
| Davies et al. (28) | 2019 | UK | Quantitative non-randomized | 80 |
| Demir et al. (29) | 2014 | UK | Quantitative non-randomized | 80 |
| Driskell et al. (30) | 2012 | UK | Quantitative non-randomized | 100 |
| Eide et al. (31) | 2012 | Norway | Quantitative descriptive | 100 |
| Elwenspoek et al. (32) | 2020 | UK | Mixed methods (quantitative descriptive + qualitative) | 40 |
| Evans et al. (33) | 2014 | UK | Quantitative non-randomized | 60 |
| Fazal et al. (34) | 2015 | UK | Quantitative non-randomized | 80 |
| Fingland et al. (35) | 2018 | UK | Quantitative non-randomized | 80 |
| Finnikin et al. (36) | 2017 | UK | Quantitative non-randomized | 80 |
| Fitzpatrick et al. (37) | 2015 | UK | Quantitative non-randomized | 80 |
| Fletcher et al. (38) | 2016 | UK | Quantitative descriptive | 100 |
| Gao et al. (39) | 2019 | UK | Quantitative non-randomized | 60 |
| Gene-Badia et al. (40) | 2016 | Spain | Qualitative studies | 100 |
| Gransjoen et al. (41) | 2018 | Norway | Qualitative studies | 100 |
| Gransjoen et al. (42) | 2020 | Norway | Qualitative studies | 100 |
| Grey et al. (43) | 2019 | UK | Quantitative non-randomized | 80 |
| Gröndal et al. (44) | 2015 | Sweden | Qualitative studies | 100 |
| Guess et al. (45) | 2015 | UK | Quantitative non-randomized | 80 |
| Gunnarsson et al. (46) | 2020 | UK | Quantitative descriptive | 80 |
| Haas et al. (47) | 2016 | USA | Quantitative descriptive | 60 |
| Hahn et al. (48) | 2021 | USA | Qualitative studies | 100 |
| Hall et al. (49) | 2019 | USA | Quantitative descriptive | 100 |
| Harkness et al. (50) | 2013 | UK | Qualitative studies | 100 |
| Hawley et al. (51) | 2016 | UK | Quantitative non-randomized | 80 |
| Healey et al. (52) | 2018 | UK | Quantitative descriptive | 100 |
| Hedin et al. (53) | 2014 | Sweden | Qualitative studies | 100 |
| Herrett et al. (54) | 2019 | UK | Quantitative non-randomized | 100 |
| Hobson et al. (55) | 2008 | UK | Quantitative non-randomized | 80 |
| Hong et al. (56) | 2016 | UK | Quantitative non-randomized | 80 |
| Hope et al. (57) | 2016 | UK | Quantitative non-randomized | 80 |
| Jameson et al. (58) | 2016 | UK | Quantitative non-randomized | 80 |
| Julian et al. (59) | 2010 | UK | Qualitative studies | 100 |
| Kelly et al. (60) | 2011 | UK | Quantitative non-randomized | 60 |
| Kendrick et al. (61) | 2015 | UK | Quantitative non-randomized | 80 |
| Khadjesari et al. (62) | 2019 | UK | Qualitative studies | 100 |
| Kidney et al. (63) | 2017 | UK | Qualitative studies | 100 |
| Kotzeva et al. (64) | 2014 | Spain | Quantitative descriptive | 80 |
| Lay-Flurrie et al. (65) | 2020 | UK | Quantitative non-randomized | 100 |
| Lay-Flurrie et al. (66) | 2021 | UK | Quantitative non-randomized | 100 |
| Le et al. (67) | 2015 | Denmark | Qualitative studies | 100 |
| Liira et al. (68) | 2015 | Finland | Qualitative studies | 100 |
| M et al. (69) | 2022 | UK | Quantitative non-randomized | 80 |
| Maclean et al. (70) | 2020 | UK | Quantitative non-randomized | 80 |
| Mc Hugh et al. (71) | 2011 | UK | Quantitative non-randomized | 60 |
| McCauley et al. (72) | 2013 | UK | Qualitative studies | 100 |
| McDonald et al. (73) | 2014 | UK | Quantitative non-randomized | 60 |
| Mejzner et al. (74) | 2017 | UK | Quantitative descriptive | 60 |
| Menon et al. (75) | 2011 | UK | Quantitative non-randomized | 80 |
| Mettias et al. (76) | 2021 | UK | Quantitative non-randomized | 80 |
| Milos et al. (77) | 2014 | Sweden | Qualitative studies | 100 |
| Mitchell et al. (78) | 2011 | UK | Qualitative studies | 100 |
| Morden et al. (79) | 2014 | UK | Qualitative studies | 100 |
| Neal et al. (80) | 2014 | UK | Quantitative non-randomized | 80 |
| Neumann et al. (81) | 2010 | Germany | Quantitative non-randomized | 60 |
| Neumark et al. (82) | 2010 | Sweden | Quantitative non-randomized | 60 |
| Nicholson et al. (83) | 2016 | UK | Quantitative descriptive | 60 |
| Nies et al. (84) | 2021 | Netherlands | Quantitative non-randomized | 60 |
| Paschalis et al. (85) | 2020 | UK | Quantitative non-randomized | 80 |
| Pate et al. (86) | 2020 | UK | Quantitative descriptive | 80 |
| Prescott et al. (87) | 2012 | UK | Quantitative non-randomized | 80 |
| Price, Spencer et al. (88) | 2020 | UK | Quantitative non-randomized | 100 |
| Price, Zhang et al. (89) | 2020 | UK | Quantitative non-randomized | 80 |
| Quyn et al. (90) | 2018 | UK | Quantitative non-randomized | 80 |
| Redaniel et al. (91) | 2015 | UK | Qualitative studies | 100 |
| Roulet et al. (92) | 2020 | UK | Quantitative descriptive | 80 |
| Roy et al. (93) | 2018 | UK | Quantitative descriptive | 80 |
| Rubin et al. (94) | 2015 | UK | Quantitative non-randomized | 60 |
| Schofield et al. (95) | 2012 | UK | Quantitative non-randomized | 100 |
| Searle et al. (96) | 2012 | UK | Qualitative studies | 100 |
| Smith et al. (97) | 2008 | UK | Quantitative non-randomized | 80 |
| Stocks et al. (98) | 2017 | UK | Quantitative non-randomized | 100 |
| Sutton et al. (99) | 2016 | UK | Quantitative descriptive | 60 |
| Taylor et al. (100) | 2023 | UK | Quantitative non-randomized | 100 |
| Thomas et al. (101) | 2022 | USA | Mixed methods (quantitative non-randomized + qualitative) | 100 |
| Tiffin et al. (102) | 2019 | UK | Quantitative non-randomized | 80 |
| Tikka et al. (103) | 2016 | UK | Quantitative non-randomized | 60 |
| Tompson et al. (104) | 2019 | UK | Qualitative studies | 100 |
| Toner et al. (105) | 2010 | UK | Quantitative descriptive | 100 |
| van Dalem et al. (106) | 2021 | UK | Quantitative non-randomized | 80 |
| Von Wagner et al. (107) | 2019 | UK | Quantitative descriptive | 80 |
| Wiering et al. (108) | 2022 | UK | Quantitative non-randomized | 80 |
| Wilkinson et al. (109) | 2018 | UK | Quantitative non-randomized | 80 |
| Wong et al. (110) | 2017 | UK | Quantitative non-randomized | 80 |
| Yamanouchi et al. (111) | 2021 | UK | Quantitative non-randomized | 100 |
| Zafar et al. (112) | 2012 | UK | Quantitative non-randomized | 60 |
| Zheng et al. (113) | 2020 | UK | Quantitative non-randomized | 80 |
| Zienius et al. (114) | 2019 | UK | Quantitative non-randomized | 100 |
| Deslandes et al. (115) | 2016 | UK | Quantitative non-randomized | 80 |
| Edwin (116) | 2014 | UK | Quantitative non-randomized | 60 |
| Eikeland et al. (117) | 2022 | Norway | Quantitative descriptive | 80 |
| Giner Galvan et al. (118) | 2020 | USA | Quantitative descriptive | 60 |
| Rian Lelie- van der Zande et al. (119) | 2021 | Netherlands | Quantitative non-randomized | 80 |
| Sperati et al. (120) | 2019 | USA | Mixed methods (quantitative descriptive + qualitative) | 80 |

**References**

1. Abdelhamid A, Howe A, Stokes T, Qureshi N, Steel N. Primary care evidence in clinical guidelines: a mixed methods study of practitioners' views. *Br J Gen Pract*. 2014 Nov;64(628):e719-27.

2. Alfirevic A, Downing J, Daras K, Comerford T, Pirmohamed M, Barr B. Has the introduction of direct oral anticoagulants (DOACs) in England increased emergency admissions for bleeding conditions? A longitudinal ecological study. *BMJ open*. 2020;10:e033357.

3. Andre M, Grondal H, Strandberg EL, Brorsson A, Hedin K. Uncertainty in clinical practice - an interview study with Swedish GPs on patients with sore throat. *BMC Fam Pract*. 2016 May 18;17(1):56.

4. Arjunan A, Jeelani MS, Docherty S, Taylor J. Chronic kidney disease referrals from general practitioners pre- and post National Institute for Health and Care Excellence guidance 2014. *Clin Med (Lond)*. 2019 Nov;19(6):490-3.

5. Bakhai M, Hopster D, Wakeel R. A retrospective study comparing the accuracy of prehistology diagnosis and surgical excision of malignant melanomas by general practitioners and hospital specialists. *Clin Exp Dermatol*. 2010 Jan;35(1):63-7.

6. Bakker L, Kemper PF, Wagner C, Delwel GO, de Bruijne MC. A baseline assessment by healthcare professionals of Dutch pharmacotherapeutic care for the elderly with polypharmacy. *Eur J Public Health*. 2017 Aug 1;27(4):679-86.

7. Barrera L, Leaper C, Pape UJ, Majeed A, Blangiardo M, Millett C. Impact of ethnic-specific guidelines for anti-hypertensive prescribing in primary care in England: a longitudinal study. *BMC Health Serv Res*. 2014 Feb 25;14(1):87.

8. Baughan P, Keatings J, O'Neill B. Urgent suspected cancer referrals from general practice: audit of compliance with guidelines and referral outcomes. *Br J Gen Pract*. 2011 Nov;61(592):e700-6.

9. Bedson J, Belcher J, Martino OI, Ndlovu M, Rathod T, Walters K, et al. The effectiveness of national guidance in changing analgesic prescribing in primary care from 2002 to 2009: an observational database study. *Eur J Pain*. 2013 Mar;17(3):434-43.

10. Bhachu HK, Cockwell P, Subramanian A, Adderley NJ, Gokhale K, Fenton A, et al. Impact of Using Risk-Based Stratification on Referral of Patients With Chronic Kidney Disease From Primary Care to Specialist Care in the United Kingdom. *Kidney Int Rep*. 2021 Aug;6(8):2189-99.

11. Bishop FL, Dima AL, Ngui J, Little P, Moss-Morris R, Foster NE, Lewith GT. "Lovely Pie in the Sky Plans": A Qualitative Study of Clinicians' Perspectives on Guidelines for Managing Low Back Pain in Primary Care in England. *Spine (Phila Pa 1976)*. 2015 Dec;40(23):1842-50.

12. Blanker MH, Koerhuis-Roessink M, Swart SJ, Zuurmond WW, van der Heide A, Perez RS, Rietjens JA. Pressure during decision making of continuous sedation in end-of-life situations in Dutch general practice. *BMC Fam Pract*. 2012 Jul 3;13(1):68.

13. Boivin JM, Tsou-Gaillet TJ, Fay R, Dobre D, Rossignol P, Zannad F. Influence of the recommendations on the implementation of home blood pressure measurement by French general practitioners: a 2004-2009 longitudinal survey. *J Hypertens*. 2011 Nov;29(11):2105-15.

14. Bottle A, Kim D, Aylin PP, Majeed FA, Cowie MR, Hayhoe B. Real-world presentation with heart failure in primary care: Do patients selected to follow diagnostic and management guidelines have better outcomes? *Open Heart*. 2018;5.

15. Brown F, Fry G, Cawood A, Stratton R. Economic Impact of Implementing Malnutrition Screening and Nutritional Management in Older Adults in General Practice. *J Nutr Health Aging*. 2020;24(3):305-11.

16. Butalid L, Verhaak PF, Bensing JM. Changes in general practitioners' sensitivity to patients' distress in low back pain consultations. *Patient Educ Couns*. 2015 Oct;98(10):1207-13.

17. Chen Y, Bedson J, Hayward RA, Jordan KP. Trends in prescribing of non-steroidal anti-inflammatory drugs in patients with cardiovascular disease: influence of national guidelines in UK primary care. *Fam Pract*. 2018 Jul 23;35(4):426-32.

18. Coulthard MG, Lambert HJ, Vernon SJ, Hunter EW, Keir MJ. Guidelines to identify abnormalities after childhood urinary tract infections: a prospective audit. *Arch Dis Child*. 2014 May;99(5):448-51.

19. Cragg J, Nyamekye I. The recommended goal in the United Kingdom's National Institute for Health and Care Excellence Clinical Guideline 168 for immediate referral of patients with bleeding varicose veins is not being achieved. *Journal of Vascular Surgery: Venous and Lymphatic Disorders*. 2021;9:377-82.

20. Crawford SM, Evans C. Outcome of elevated CA125 values from primary care following implementation of ovarian cancer guidelines. *Fam Pract*. 2018 Mar 27;35(2):199-202.

21. Cubiella J, Salve M, Diaz-Ondina M, Vega P, Alves MT, Iglesias F, et al. Diagnostic accuracy of the faecal immunochemical test for colorectal cancer in symptomatic patients: comparison with NICE and SIGN referral criteria. *Colorectal Dis*. 2014 Aug;16(8):O273-82.

22. Curtis HJ, Walker AJ, Goldacre B. Impact of NICE guidance on tamoxifen prescribing in England 2011–2017: an interrupted time series analysis. *British journal of cancer*. 2018;118(9):1268-75.

23. Cuvellier JC, Carvalho S, Mars A, Auvin S. Study on management of pediatric migraine by general practitioners in northern France. *J Headache Pain*. 2009 Jun;10(3):167-75.

24. Czarnawska-Iliev I, Robinson N. General Practitioners' use of and attitudes to acupuncture in relation to the UK's National Institute for Health and Care Excellence (NICE) clinical guidelines-A pilot study. *European Journal of Integrative Medicine*. 2016 Aug;8(4):342-54.

25. Damery S, Ryan R, Wilson S, Ismail T, Hobbs R, Improving Colorectal Outcomes G. Iron deficiency anaemia and delayed diagnosis of colorectal cancer: a retrospective cohort study. *Colorectal Dis*. 2011 Apr;13(4):e53-60.

26. Davies HOB, Popplewell M, Bate G, Ryan RP, Marshall TP, Bradbury AW. Analysis of Effect of National Institute for Health and Care Excellence Clinical Guideline CG168 on Management of Varicose Veins in Primary Care Using the Health Improvement Network Database. *Eur J Vasc Endovasc Surg*. 2018 Dec;56(6):880-4.

27. Davies HO, Popplewell M, Bate G, Kelly L, Koutsoumpelis A, Bradbury AW. Impact of UK NICE Clinical Guidelines 168 and social deprivation on access to interventional treatment for symptomatic varicose vein and specialist referral for leg ulceration. *Phlebology*. 2017 Sep;32(8):548-52.

28. Davies HOB, Popplewell M, Bate G, Ryan RP, Marshall TP, Bradbury AW. Publication of UK NICE Clinical Guidelines 168 has not significantly changed the management of leg ulcers in primary care: An analysis of The Health Improvement Network database. *Phlebology*. 2019;34(5):311-6.

29. Demir OM, Dobson P, Shaw A, Khan J, Papamichael ND, Byrne J, Alfakih K. A comparison of ESC and NICE guidelines for patients with suspected CAD: evaluation of the pre-test probability risk scores in clinical practice. *European Heart Journal*. 2014 Sep 1;35:1057-.

30. Driskell OJ, Holl, D., Hanna FW, Jones PW, Pemberton RJ, et al. Inappropriate requesting of glycated hemoglobin (Hb A1c) is widespread: Assessment of prevalence, impact of national guidance, and practice-To-practice variability. *Clinical Chemistry*. 2012;58:906-15.

31. Eide TB, Hippe VC, Brekke M. The feasibility of antibiotic dosing four times per day: a prospective observational study in primary health care. *Scand J Prim Health Care*. 2012 Mar;30(1):16-20.

32. Elwenspoek MMC, Mann E, Alsop K, Clark H, Patel R, Watson JC, Whiting P. GP's perspectives on laboratory test use for monitoring long-term conditions: an audit of current testing practice. *BMC Fam Pract*. 2020 Dec 5;21(1):257.

33. Evans DG, Brentnall AR, Harvie M, Dawe S, Sergeant JC, Stavrinos P, et al. Breast cancer risk in young women in the national breast screening programme: implications for applying NICE guidelines for additional screening and chemoprevention. *Cancer Prev Res (Phila)*. 2014 Oct;7(10):993-1001.

34. Fazal IA, Bhagra SK, Bailey KM, Dermot Neely R, MacGowan GA, Skinner JS. Impact of using different guideline recommended serum natriuretic peptide thresholds on the diagnosis and referral rates of a diagnostic heart failure clinic. *Int J Clin Pract*. 2015 Nov;69(11):1349-56.

35. Fingland P, Carswell V, Tikka T, Douglas CM, Montgomery J. The value of chest X-ray in the Scottish Referral Guidelines for suspected head and neck cancer in 2144 patients. *J Laryngol Otol*. 2018 May;132(5):434-8.

36. Finnikin S, Ryan R, Marshall T. Statin initiations and QRISK2 scoring in UK general practice: a THIN database study. *Br J Gen Pract*. 2017 Dec;67(665):e881-e7.

37. Fitzpatrick RW, Pate RG. Assessing the impact of NICE guidance on the prescribing of hormonal treatments of breast cancer in England. *J Eval Clin Pract*. 2015 Aug;21(4):759-61.

38. Fletcher BR, Hinton L, Bray EP, Hayen A, Hobbs FR, Mant J, et al. Self-monitoring blood pressure in patients with hypertension: an internet-based survey of UK GPs. *Br J Gen Pract*. 2016 Nov;66(652):e831-e7.

39. Gao C, Qin C, Freeman S, Oskooee N, Hughes J. Two week wait referral criteria–heading in the right direction? *The Journal of Laryngology & Otology*. 2019;133(8):704-12.

40. Gene-Badia J, Gallo P, Cais J, Sanchez E, Carrion C, Arroyo L, Aymerich M. The use of clinical practice guidelines in primary care: professional mindlines and control mechanisms. *Gac Sanit*. 2016 Sep-Oct;30(5):345-51.

41. Gransjoen AM, Wiig S, Lysdahl KB, Hofmann BM. Barriers and facilitators for guideline adherence in diagnostic imaging: an explorative study of GPs' and radiologists' perspectives. *BMC Health Serv Res*. 2018 Jul 16;18(1):556.

42. Gransjoen AM, Wiig S, Lysdahl KB, Hofmann BM. Health care personnel's perception of guideline implementation for musculoskeletal imaging: a process evaluation. *BMC Health Serv Res*. 2020 May 11;20(1):397.

43. Grey R, Walsh S. A review of the electronic two-week rule referrals for head and neck cancer to Western Sussex Hospitals NHS Foundation Trust. *Br Dent J*. 2019 Sep;227(5):403-9.

44. Gröndal H, Hedin K, Strandberg EL, André M, Brorsson A. Near-patient tests and the clinical gaze in decision-making of Swedish GPs not following current guidelines for sore throat–a qualitative interview study. *BMC family practice*. 2015;16(1):1-7.

45. Guess ND, Caengprasath N, Dornhorst A, Frost GS. Adherence to NICE guidelines on diabetes prevention in the UK: Effect on patient knowledge and perceived risk. *Prim Care Diabetes*. 2015 Dec;9(6):407-11.

46. Gunnarsson R, Ebell MH, Wachtler H, Manchal N, Reid L, Malmberg S, et al. Association between guidelines and medical practitioners' perception of best management for patients attending with an apparently uncomplicated acute sore throat: a cross-sectional survey in five countries. *BMJ Open*. 2020 Sep 17;10(9):e037884.

47. Haas JS, Sprague BL, Klabunde CN, Tosteson AN, Chen JS, Bitton A, et al. Provider Attitudes and Screening Practices Following Changes in Breast and Cervical Cancer Screening Guidelines. *J Gen Intern Med*. 2016 Jan;31(1):52-9.

48. Hahn EE, Munoz-Plaza CE, Lee EA, Luong TQ, Mittman BS, Kanter MH, et al. Patient and Physician Perspectives of Deprescribing Potentially Inappropriate Medications in Older Adults with a History of Falls: a Qualitative Study. *J Gen Intern Med*. 2021 Oct;36(10):3015-22.

49. Hall TL, Knierim KE, Nease DE, Jr., Staton EW, Nkouaga C, Miriam Dickinson L, et al. Primary Care Practices' Implementation of Patient-Team Partnership: Findings from EvidenceNOW Southwest. *J Am Board Fam Med*. 2019 Jul-Aug;32(4):490-504.

50. Harkness EF, Harrington V, Hinder S, O'Brien SJ, Thompson DG, Beech P, Chew-Graham CA. GP perspectives of irritable bowel syndrome--an accepted illness, but management deviates from guidelines: a qualitative study. *BMC Fam Pract*. 2013 Jun 27;14:92.

51. Hawley S, Leal J, Delmestri A, Prieto-Alhambra D, Arden NK, Cooper C, et al. Anti-Osteoporosis Medication Prescriptions and Incidence of Subsequent Fracture Among Primary Hip Fracture Patients in England and Wales: An Interrupted Time-Series Analysis. *J Bone Miner Res*. 2016 Nov;31(11):2008-15.

52. Healey EL, Afolabi EK, Lewis M, Edwards JJ, Jordan KP, Finney A, et al. Uptake of the NICE osteoarthritis guidelines in primary care: a survey of older adults with joint pain. *BMC Musculoskelet Disord*. 2018 Aug 17;19(1):295.

53. Hedin K, Strandberg EL, Grondal H, Brorsson A, Thulesius H, Andre M. Management of patients with sore throats in relation to guidelines: an interview study in Sweden. *Scand J Prim Health Care*. 2014 Dec;32(4):193-9.

54. Herrett E, Gadd S, Jackson R, Bhaskaran K, Williamson E, van Staa T, et al. Eligibility and subsequent burden of cardiovascular disease of four strategies for blood pressure-lowering treatment: a retrospective cohort study. *Lancet*. 2019 Aug 24;394(10199):663-71.

55. Hobson JC, Malla JV, Sinha J, Kay NJ, Ramamurthy L. Outcomes for patients referred urgently with suspected head and neck cancer. *J Laryngol Otol*. 2008 Nov;122(11):1241-4.

56. Hong B, Shaikh Z, Adcock S, Aldallal SN. Two-week wait false alarms? A prospective investigation of 2WW head and neck cancer referrals. *Br Dent J*. 2016 May 27;220(10):521-6.

57. Hope SV, Wienand-Barnett S, Shepherd M, King SM, Fox C, Khunti K, et al. Practical Classification Guidelines for Diabetes in patients treated with insulin: a cross-sectional study of the accuracy of diabetes diagnosis. *Br J Gen Pract*. 2016 May;66(646):e315-22.

58. Jameson K, D'Oca K, Leigh P, Murray-Thomas T. Adherence to NICE guidance on glucagon-like peptide-1 receptor agonists among patients with type 2 diabetes mellitus: an evaluation using the Clinical Practice Research Datalink. *Curr Med Res Opin*. 2016 Jan 2;32(1):49-60.

59. Julian S, Rashid A, Baker R, Szczepura A, Habiba M. Attitudes of women with menstrual disorders to the use of clinical guidelines in their care. *Fam Pract*. 2010 Apr;27(2):205-11.

60. Kelly D, Cole S, Rossiter F, Mallinson K, Smith A, Simpson I. Implementation of the new NICE guidelines for stable chest pain: likely impact on chest pain services in the UK. *British Journal of Cardiology*. 2011;18(4):185-8.

61. Kendrick T, Stuart B, Newell C, Geraghty AW, Moore M. Did NICE guidelines and the Quality Outcomes Framework change GP antidepressant prescribing in England? Observational study with time trend analyses 2003–2013. *Journal of Affective Disorders*. 2015;186:171-7.

62. Khadjesari Z, Stevenson F, Toner P, Linke S, Milward J, Murray E. 'I'm not a real boozer': a qualitative study of primary care patients' views on drinking and its consequences. *J Public Health (Oxf)*. 2019 Jun 1;41(2):e185-e91.

63. Kidney E, Greenfield S, Berkman L, Dowswell G, Hamilton W, Wood S, Marshall T. Cancer suspicion in general practice, urgent referral, and time to diagnosis: A population-based GP survey nested within a feasibility study using information technology to flag-up patients with symptoms of colorectal cancer. *BJGP Open*. 2017;1(3).

64. Kotzeva A, Guillamon I, Gracia J, Diaz del Campo P, Gich I, Calderon E, et al. Use of clinical practice guidelines and factors related to their uptake: a survey of health professionals in Spain. *J Eval Clin Pract*. 2014 Jun;20(3):216-24.

65. Lay-Flurrie SL, Sheppard JP, Stevens RJ, Mallen C, Heneghan C, Hobbs FDR, et al. Impact of Changes to National Hypertension Guidelines on Hypertension Management and Outcomes in the United Kingdom. *Hypertension*. 2020 Feb;75(2):356-64.

66. Lay-Flurrie SL, Sheppard JP, Stevens RJ, Mallen C, Heneghan C, Hobbs FR, et al. Impact of changes to national guidelines on hypertension-related workload: an interrupted time series analysis in English primary care. *Br J Gen Pract*. 2021 Apr;71(705):e296-e302.

67. Le JV, Hansen HP, Riisgaard H, Lykkegaard J, Nexoe J, Bro F, Sondergaard J. How GPs implement clinical guidelines in everyday clinical practice--a qualitative interview study. *Fam Pract*. 2015 Dec;32(6):681-5.

68. Liira H, Saarelma O, Callaghan M, Harbour R, Jousimaa J, Kunnamo I, et al. Patients, health information, and guidelines: A focus-group study. *Scand J Prim Health Care*. 2015;33(3):212-9.

69. M C, Ic C, S S. Impact of the updated NICE referral pathway for patients with suspected brain cancer on a neuroscience service. *Br J Neurosurg*. 2022 Feb;36(1):11-5.

70. Maclean W, Singh R, Mackenzie P, White D, Benton S, Stebbing J, et al. The two-week rule colorectal cancer pathway: an update on recent practice, the unsustainable burden on diagnostics and the role of faecal immunochemical testing. *Ann R Coll Surg Engl*. 2020 Apr;102(4):308-11.

71. Mc Hugh S, Marsden P, Brennan C, Murphy K, Croarkin C, Moran J, et al. Counting on commitment; the quality of primary care-led diabetes management in a system with minimal incentives. *BMC Health Serv Res*. 2011 Dec 28;11:348.

72. McCauley CO, Casson K. A qualitative study into how guidelines facilitate general practitioners to empower women to make decisions regarding antidepressant use in pregnancy. *International Journal of Mental Health Promotion*. 2013;15(1):3-28.

73. McDonald K, Kenney I. Paediatric urinary tract infections: a retrospective application of the National Institute of Clinical Excellence guidelines to a large general practitioner referred historical cohort. *Pediatr Radiol*. 2014 Sep;44(9):1085-92.

74. Mejzner N, Clark CE, Smith LF, Campbell JL. Trends in the diagnosis and management of hypertension: repeated primary care survey in South West England. *Br J Gen Pract*. 2017 May;67(658):e306-e13.

75. Menon R, Larner AJ. Use of cognitive screening instruments in primary care: the impact of national dementia directives (NICE/SCIE, National Dementia Strategy). *Fam Pract*. 2011 Jun;28(3):272-6.

76. Mettias B, Charlton A, Ashokkumar S. Outcome of two-week head and neck cancer pathway for the otolaryngology department in a tertiary centre. *J Laryngol Otol*. 2021 Oct;135(10):869-73.

77. Milos V, Westerlund T, Midlov P, Strandberg EL. Swedish general practitioners' attitudes towards treatment guidelines - a qualitative study. *BMC Fam Pract*. 2014 Dec 16;15(1):199.

78. Mitchell C, Dwyer R, Hagan T, Mathers N. Impact of the QOF and the NICE guideline in the diagnosis and management of depression: a qualitative study. *Br J Gen Pract*. 2011 May;61(586):e279-89.

79. Morden A, Jinks C, Ong BN, Porcheret M, Dziedzic KS. Acceptability of a 'guidebook' for the management of Osteoarthritis: a qualitative study of patient and clinician's perspectives. *BMC Musculoskelet Disord*. 2014 Dec 13;15(1):427.

80. Neal RD, Din NU, Hamilton W, Ukoumunne OC, Carter B, Stapley S, Rubin G. Comparison of cancer diagnostic intervals before and after implementation of NICE guidelines: analysis of data from the UK General Practice Research Database. *Br J Cancer*. 2014 Feb 4;110(3):584-92.

81. Neumann A, Jahn R, Diehm C, Driller E, Hessel F, Lux G, et al. Outcomes of medical management of peripheral arterial disease in general practice: Follow-up results of the PACE-PAD Study. *Journal of Public Health*. 2010;18:523-32.

82. Neumark T, Brudin L, Molstad S. Use of rapid diagnostic tests and choice of antibiotics in respiratory tract infections in primary healthcare--a 6-y follow-up study. *Scand J Infect Dis*. 2010;42(2):90-6.

83. Nicholson BD, Mant D, Neal RD, Hart N, Hamilton W, Shinkins B, et al. International variation in adherence to referral guidelines for suspected cancer: a secondary analysis of survey data. *Br J Gen Pract*. 2016 Feb;66(643):e106-13.

84. Nies LM, Akker ILD, Rozendaal L, Baar B, Vos RC, Hart HE. The impact of the new Dutch guideline on cardiovascular risk management in patients with COPD: a retrospective study. *BJGP Open*. 2021 Jan;5(1):1-10.

85. Paschalis T, Jones C. Plasma HbA1c in the investigation of suspected heart failure in general practice: An audit of the 2018 NICE guidelines update. *J Family Med Prim Care*. 2020 Feb;9(2):1098-102.

86. Pate A, Emsley R, van Staa T. Impact of lowering the risk threshold for statin treatment on statin prescribing: a descriptive study in English primary care. *Br J Gen Pract*. 2020 Nov;70(700):e765-e71.

87. Prescott A, Bailey JE, Kelly KJ, Munyombwe T, Gray A, Summers LK. The effectiveness and cost of single and multi-factorial cardiovascular risk factor modification to guideline targets in type 2 diabetes. *Prim Care Diabetes*. 2012 Apr;6(1):67-73.

88. Price S, Spencer A, Zhang X, Ball S, Lyratzopoulos G, Mujica-Mota R, et al. Trends in time to cancer diagnosis around the period of changing national guidance on referral of symptomatic patients: A serial cross-sectional study using UK electronic healthcare records from 2006-17. *Cancer Epidemiol*. 2020 Dec;69:101805.

89. Price S, Zhang X, Spencer A. Measuring the impact of national guidelines: What methods can be used to uncover time-varying effects for healthcare evaluations? *Social Science & Medicine*. 2020;258:N.PAG-N.PAG.

90. Quyn AJ, Steele RJ, Digby J, Strachan JA, Mowat C, McDonald PJ, et al. Application of NICE guideline NG12 to the initial assessment of patients with lower gastrointestinal symptoms: not FIT for purpose? *Ann Clin Biochem*. 2018 Jan;55(1):69-76.

91. Redaniel MT, Ridd M, Martin RM, Coxon F, Jeffreys M, Wade J. Rapid diagnostic pathways for suspected colorectal cancer: views of primary and secondary care clinicians on challenges and their potential solutions. *BMJ Open*. 2015 Oct 22;5(10):e008577.

92. Roulet C, Rozsnyai Z, Jungo KT, M AvdP, Floriani C, Kurpas D, et al. Managing hypertension in frail oldest-old-The role of guideline use by general practitioners from 29 countries. *PLoS One*. 2020;15(7):e0236064.

93. Roy S, Anjum K. The two-week wait - a qualitative analysis of suspected head and neck cancer referrals. *Br Dent J*. 2018 Jul 27;225(2):159-63.

94. Rubin GP, Saunders CL, Abel GA, McPhail S, Lyratzopoulos G, Neal RD. Impact of investigations in general practice on timeliness of referral for patients subsequently diagnosed with cancer: analysis of national primary care audit data. *Br J Cancer*. 2015 Feb 17;112(4):676-87.

95. Schofield P, Baawuah F, Seed PT, Ashworth M. Managing hypertension in general practice: a cross-sectional study of treatment and ethnicity. *Br J Gen Pract*. 2012 Oct;62(603):e703-9.

96. Searle A, Calnan M, Turner KM, Lawlor DA, Campbell J, Chalder M, Lewis G. General practitioners' beliefs about physical activity for managing depression in primary care. *Mental Health and Physical Activity*. 2012;5(1):13-9.

97. Smith CJ, Gribbin J, Challen KB, Hubbard RB. The impact of the 2004 NICE guideline and 2003 General Medical Services contract on COPD in primary care in the UK. *QJM*. 2008 Feb;101(2):145-53.

98. Stocks SJ, Kontopantelis E, Webb RT, Avery AJ, Burns A, Ashcroft DM. Antipsychotic Prescribing to Patients Diagnosed with Dementia Without a Diagnosis of Psychosis in the Context of National Guidance and Drug Safety Warnings: Longitudinal Study in UK General Practice. *Drug Saf*. 2017 Aug;40(8):679-92.

99. Sutton J, Melia J, Kirby M, Graffy J, Moss S. GPs views and understanding of PSA testing, screening and early detection; survey. *Int J Clin Pract*. 2016 May;70(5):389-95.

100. Taylor CJ, Ordonez-Mena JM, Lay-Flurrie SL, Goyder CR, Taylor KS, Jones NR, et al. Natriuretic peptide testing and heart failure diagnosis in primary care: diagnostic accuracy study. *Br J Gen Pract*. 2023 Jan;73(726):e1-e8.

101. Thomas TW, Golin CE, Kinlaw AC, Kirkman MS, Golden SD, Lightfoot AF, Samuel-Hodge CD. Did the 2015 USPSTF Abnormal Blood Glucose Recommendations Change Clinician Attitudes or Behaviors? A Mixed-Method Assessment. *J Gen Intern Med*. 2022 Jan;37(1):15-22.

102. Tiffin PA, Mediavilla JL, Close H, Kasim AS, Welsh P, Paton LW, Mason JM. What were the impacts of the Committee on Safety of Medicines warning and publication of the NICE guidelines on trends in child and adolescent antidepressant prescribing in primary care? A population based study. *BMJ Open*. 2019;9.

103. Tikka T, Pracy P, Paleri V. Refining the head and neck cancer referral guidelines: a two centre analysis of 4715 referrals. *British Journal of Oral and Maxillofacial Surgery*. 2016;54(2):141-50.

104. Tompson A, Nicholson BD, Ziebland S, Evans J, Bankhead C. Quality improvements of safety-netting guidelines for cancer in UK primary care: insights from a qualitative interview study of GPs. *Br J Gen Pract*. 2019 Dec;69(689):e819-e26.

105. Toner R, Snape C, Acton S, Blenkiron P. Do general practitioners adhere to NICE guidelines for depression? Systematic questionnaire survey. *Primary Health Care Research and Development*. 2010;11(2):123-31.

106. van Dalem J, Brouwers MCGJ, Burden AM, Stehouwer CDA, Klungel OH, de Vries F, et al. Determinants of treatment modification before and after implementation of the updated 2015 NICE guideline on type 2 diabetes: A retrospective cohort study. *Diabetes Research & Clinical Practice*. 2021;176:N.PAG-N.PAG.

107. Von Wagner C, Stoffel ST, Freeman M, Laszlo HE, Nicholson BD, Sheringham J, et al. General practitioners' awareness of the recommendations for faecal immunochemical tests (FITs) for suspected lower gastrointestinal cancers: a national survey. *BMJ Open*. 2019 Apr 11;9(4):e025737.

108. Wiering B, Lyratzopoulos G, Hamilton W, Campbell J, Abel G. Concordance with urgent referral guidelines in patients presenting with any of six 'alarm' features of possible cancer: a retrospective cohort study using linked primary care records. *BMJ Qual Saf*. 2022 Aug;31(8):579-89.

109. Wilkinson S, Douglas I, Stirnadel-Farrant H, Fogarty D, Pokrajac A, Smeeth L, Tomlinson L. Changing use of antidiabetic drugs in the UK: trends in prescribing 2000-2017. *BMJ Open*. 2018 Jul 28;8(7):e022768.

110. Wong BYW, Fischer S, Cruickshank HE. Clinical outcome of head and neck cancer patients: a comparison between ENT patients referred via the 2 weeks wait pathway and alternative routes in the UK health system. *Eur Arch Otorhinolaryngol*. 2017 Jan;274(1):415-20.

111. Yamanouchi L, Srinivasan M, Barlow N, Basu A. Level of adherence to vitamin D supplementation guidelines in an antenatal centre in Birmingham, UK, and its effect on biochemical and obstetrical outcomes: a single-centre cross-sectional study. *BMJ Open*. 2021 Sep 15;11(9):e048705.

112. Zafar A, Mak T, Whinnie S, Chapman M. The 2‐week wait referral system does not improve 5‐year colorectal cancer survival. *Colorectal Disease*. 2012;14(4):e177-e80.

113. Zheng A, Cowan E, Mach L, Adam RD, Guha K, Cowburn PJ, et al. Characteristics and outcomes of patients with suspected heart failure referred in line with National Institute for Health and Care Excellence guidance. *Heart*. 2020 Oct;106(20):1579-85.

114. Zienius K, Chak-Lam I, Park J, Ozawa M, Hamilton W, Weller D, et al. Direct access CT for suspicion of brain tumour: an analysis of referral pathways in a population-based patient group. *BMC Fam Pract*. 2019 Aug 20;20(1):118.

115. Desl, es PN, Jenkins KSL, Haines KE, Hutchings S, Cannings‐John R, et al. A change in the trend in dosulepin usage following the introduction of a prescribing indicator but not after two national safety warnings. *Journal of Clinical Pharmacy & Therapeutics*. 2016;41(2):224-8.

116. Edwin MF. Adult ADHD outcome audit based on NICE guidelines. *Advances in Mental Health and Intellectual Disabilities*. 2014;8(5):331-7.

117. Eikeland SA, Smeland KB, Brekke M, Kiserud CE, Fossa A. Late-effect awareness and follow-up of cancer in general practice. *Scand J Prim Health Care*. 2022 Sep;40(3):360-9.

118. Giner Galvan V, Bonig Trigueros I, Facila Rubio L, Morillas Blasco P, Martinez Hervas S, Pascual Fuster V, et al. Have the Government's prescription algorithm and the 2013 American College of Cardiology/American Heart Association guidelines for managing dyslipidaemia influenced the management of dyslipidaemia? The MEJORALO-CV Project. *Revista Clinica Espanola*. 2020;220:282-9.

119. Z L-vd, e R, Bouvy M, Teichert M. Adherence to guideline recommendations for urinary tract infections in adult women: a cross-sectional study. *Primary health care research & development*. 2021;22:e11.

120. John Sperati C, Soman S, Agrawal V, Liu Y, Abdel-Kader K, Diamantidis CJ, et al. Primary care physicians' perceptions of barriers and facilitators to management of chronic kidney disease: A mixed methods study. *PLoS ONE*. 2019;14.
