# Supplementary material for: Outcomes of guidelines from health technology assessment organizations in community-based primary care: a systematic mixed studies review
Source: Int J Technol Assess Health Care. 2024 Nov 14;40(1):e56. doi: 10.1017/S0266462324000370 (PMC11579698; doi:10.1017/S0266462324000370)
Supplement: Baradaran et al. supplementary material [file S0266462324000370sup001.zip › Appendix 7.docx]

| **Appendix 7.** MMAT results. Numbers are representing the count of studies in each section. | | | | | | |
| --- | --- | --- | --- | --- | --- | --- |
| **Study type** | **Results** | | | | | |
|  | **100%** | **80%** | **60%** | **40%** | **20%** | **0%** |
| All included studies (n=120) | 44 | 55 | 20 | 1 |  |  |
| Qualitative studies and qualitative components of mixed-methods studies (n=26) | 26 |  |  |  |  |  |
| Quantitative studies and quantitative components of mixed-methods studies (n=99) | 23 | 55 | 20 | 1 |  |  |
