# Supplementary material for: Outcomes of guidelines from health technology assessment organizations in community-based primary care: a systematic mixed studies review
Source: Int J Technol Assess Health Care. 2024 Nov 14;40(1):e56. doi: 10.1017/S0266462324000370 (PMC11579698; doi:10.1017/S0266462324000370)
Supplement: Baradaran et al. supplementary material [file S0266462324000370sup001.zip › Appendix 8.docx]

**
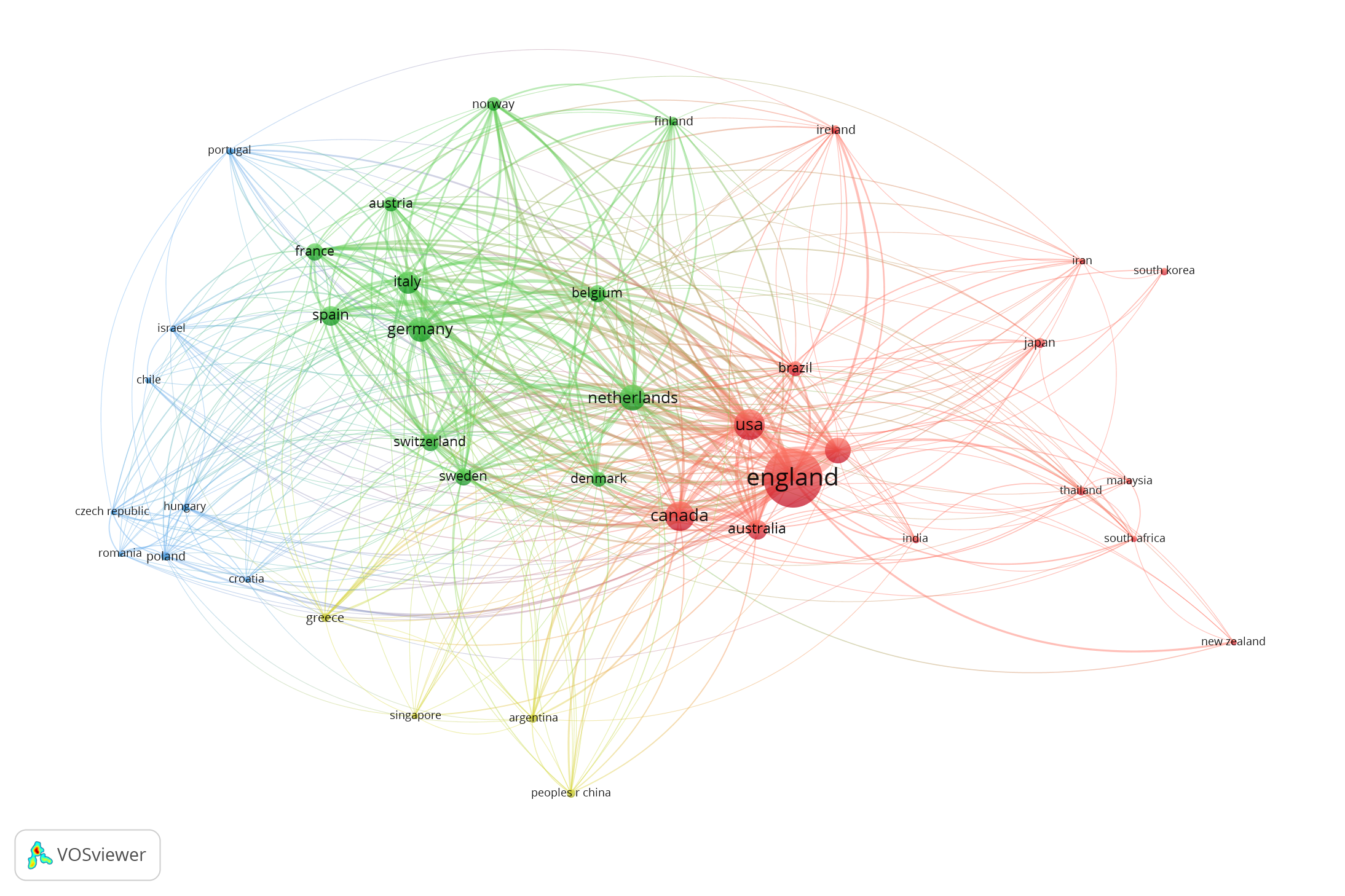
Appendix 8.** Bibliometric study of authors’ affiliations. A preliminary search was done on the Web of Science and authors' affiliations were extracted and displayed using VOSviewer. Co-authorships are shown in color. From what can be seen, the majority of the authors of publications on our subject were affiliated with institutes in England.
